# Increasing engagement in price crowdsourcing initiatives: Using nudges in Nigeria

**DOI:** 10.1016/j.worlddev.2022.105818

**Published:** 2022-04

**Authors:** Gloria Solano-Hermosilla, Jesus Barreiro-Hurle, Julius B. Adewopo, Celso Gorrín-González

**Affiliations:** a Joint Research Centre, European Commission, Spain; b International Institute for Tropical Agriculture (IITA), Nigeria

**Keywords:** Social norms, Information disclosure, Nudges, Crowdsourcing, Food market prices, Food security

## Abstract

African agriculture is yet to reach its full food production potential. One way of addressing this is a better distribution of market signals to farmers and other market participants, which can help them make better-informed decisions, leading to increased income and capacity for investment. Hence, increasing the availability of market information in Africa is a priority and alternative data sources, and new Information Communication Technologies (ICTs) offer huge potential to complement classic official statistics. This has given rise to a number of ICTs and citizen science projects to monitor data in real time, of which food price crowdsourcing in Africa is one. However, one of the main challenges faced by crowdsourcing initiatives is to ensure that individuals feed useful information into the system. In this paper, we test the potential of behavioural interventions to help sustain crowd contributions by leveraging intrinsic and/or extrinsic motivations. We used two randomised control trials (RCTs) to evaluate whether the inclusion of two nudges (one based on social norms and one based on information disclosure) in the design of a food price crowdsourcing initiative can improve crowd engagement. Our results show that social norms increase crowd participation while disclosing price information does not. The latter highlights the need for further research to identify which type of information and format to make it accessible would best help to sustain crowd effort levels. These findings have the potential to be useful in designing future crowdsourcing (or other types of) initiatives that require sustained citizen engagement over time.

## Highlights

• Crowdsourcing initiatives can complement official price statistics and help to create better functioning food markets in Africa.
• Sustaining crowd engagement in crowdsourcing initiatives remains a challenge for the institutions implementing them.
• We used RCTs to test the potential of social norms and information disclosure to sustain crowd engagement in a food price crowdsourcing initiative in Nigeria.
• Interventions using social norms increased the number of submissions.
• Interventions using information disclosure failed to increase the number of submissions.

## 1 Introduction

African agriculture is yet to reach its full food production potential (AGRA, 2017). One of the reasons for the lag in Africa’s food production is the imperfect distribution of market signals to farmers which prevents them from making informed decisions that could maximise their income and lead to increased welfare and the capacity to invest (Short et al., 2014). Therefore, increasing the availability of market information in Africa has become a priority for both national statistical offices and development partners. One of the most recent efforts to provide food price information focuses on the use of crowdsourcing methods based on citizen engagement and technology-enabled solutions (Zeug et al., 2017). Despite subtle differences in how it is conceptualised, crowdsourcing is commonly defined as a participatory method of obtaining data or inputs from a number of people who are not paid employees (the crowd) and using ICTs (Brabham, 2013, Estellés-Arolas and González-Ladrón-De-Guevara, 2012). Rather than a substitute for traditional statistical approaches, crowdsourcing has the potential to complement these and help bridge data gaps in terms of timeliness, frequency, and commodity or geographical scope.

However, one of the main challenges faced by crowdsourcing initiatives is the need to ensure that individuals feed information into the system, and that this information is complete and accurate. This becomes particularly relevant for initiatives relying on unrewarded voluntary contributions, or which discontinue rewards after the initial phase, a common feature of donor-sponsored initiatives. Experience shows that most crowdsourcing initiatives cannot sustain voluntary input once the funding source for rewards expires. This is because participants’ and users’ motivation, rather than technology, is the key constraint on effective crowdsourcing (Ziegler et al., 2017).

Beyond economic incentives, behavioural science can provide tools to help sustain crowd contributions by activating levers that can strengthen individuals’ engagement with the platform. These factors can be integrated into the design of crowdsourcing tools to maximise the quantity of data gathered. In this paper, we report the results of a placebo-controlled field trial where we test behavioural informed interventions to sustain crowd effort.

In particular, we focus on the ability of nudges to affect the number of submissions to a crowdsourcing initiative. Nudges are defined as ‘liberty-preserving approaches that steer people in particular directions’ (Sunstein, 2014). In short, a nudge is an action (i.e. providing information, changing the setting of a decision) that can activate behaviours that lead people to act in a certain way. In this sense, nudges offer a cheap and effective way of influencing citizens’ behaviour, without restricting freedom of choice, imposing mandatory obligations, or providing monetary incentives. In our context, the most important task for nudges is to enhance the potential of information provision as a trigger for action (Ölander & Thøgersen, 2014). Monetary rewards activate what researchers on crowd motivational factors call ‘*extrinsic motivation’,* i.e. factors coming from outside the individual, and providing an immediate or delayed payoff. By contrast, nudges are expected to activate *intrinsic motivation;* intangible motivation coming from within the individual, and linked to personal satisfaction or accomplishment, for example, ‘fun’, ‘enjoyment’, ‘learning’, ‘social interaction’, and ‘social contribution’ (Kaufmann et al., 2011, Pedersen et al., 2013).

We evaluate the impact of two types of nudges on crowd engagement by implementing two randomised controlled trials (RCTs) as part of a crowdsourcing initiative in Nigeria: one based on social norms, and another based on information disclosure. The nudges are implemented in addition to a first-come, first-payed (up to a daily limit) financial reward system. Social norms can be defined as ‘‘rules and standards that are understood by members of a group, and that guide and/or constrain social behaviour without the force of law’’ (Cialdini & Trost, 1998). In particular, we focus on the use of descriptive norms, communicating to the crowd what others do (Bobek et al., 2007). Nudges using descriptive norms have proven to be effective in multiple interventions. These range from increasing tax compliance (Cullis et al., 2012) and retirement savings (Benartzi and Thaler, 2007, Madrian and Shea, 2001), to child school attendance (Damgaard & Nielsen, 2018), waste recycling (Linder et al., 2018), healthy eating (Kroese et al., 2015), sustainable consumption (Demarque et al., 2015), and household action on climate change (Nisa et al., 2019).

The second type of nudge tested the potential to change behaviour via ‘collectively gathered information disclosure’ (Loewenstein et al., 2014). For example, Motoshita et al. (2015) found that providing quantitative information on the carbon dioxide emissions of different shopping methods affected consumers choices of food and drinks. Also, providing information on drinking water standards violations led to a significant reduction in non-compliance with standards (Bennear & Olmstead, 2008). We provide crowd members with a link to access a data dashboard with different indicators constructed with the contributions of the crowd. This nudge mobilises different intrinsic motivational factors related to social duty, for instance, if the person sees their price submissions as a contribution to a ‘common good’, they are more likely to participate in the data sourcing exercise. It can also activate the utilitarian and hedonic perspectives of crowdsourcing systems (Soliman and Tuunainen, 2015, Wu and Lu, 2013). The way information is disclosed or provided (e.g. through interactive dashboards) could raise other intrinsic motivational factors, such as enjoyment or pleasure (hedonic perspective) in the use of the crowdsourcing system (Soliman and Tuunainen, 2015, Wu and Lu, 2013).

Furthermore, if the crowd participants can use the collective crowdsourced information in making decisions, disclosing crowdsourced information could also foster improved decision making, while respecting their choice (Loewenstein et al., 2014). Previous literature shows that price information sharing can improve market integration by enhancing spatial arbitrage and efficiency by reducing information asymmetries and increasing farmers' bargaining power, eventually empowering farmers to obtain higher prices from intermediaries (Jensen, 2010, Pendell and Schroeder, 2006), especially when barriers to switching are low (Mitchell, 2017). However, this extrinsic motivation to contribute based on self-utility may be reduced if people consider they can enjoy the benefits of the effort of others, without contributing themselves (‘free riding’). Moreover, the extrinsic motive would not be activated if participants could not access this information due to technical or connectivity problems.

Previous research on crowdsourcing finds that the crowd responds to material or extrinsic incentives, but also to other motivational factors related to internal satisfaction (Blaschke et al., 2013). To the best of our knowledge, nudges have not been extensively tested in the field of crowdsourcing of food prices, the exception being Blaschke et al. (2013). Our study makes an important contribution to research by bringing together two streams of literature; that on behavioural science, and that on crowdsourcing systems. We provide empirical evidence of the effects of motivational factors on people’s propensity to contribute to the crowdsourcing data initiative. Our results show that social norms have an impact on submissions while information disclosure fails to activate the expected behaviour, probably due to the lack of user friendliness of the dissemination format used. From a practical perspective, the results can help guide current and future crowdsourcing efforts.

Despite the fact that our experiment was not pre-registered, the main hypothesis (both interventions having a positive impact on submissions) and the statistical analysis performed (difference in difference applied to a randomized control trial) are straightforward and drove the design of the experiment we report. However, the additional analysis reported should be considered exploratory.

The paper is structured as follows: Section 2 describes the data, the experimental design for evaluating the performance of the nudges, theoretical backing of the hypotheses tested, and method of analysis, Section 3 presents the results and discussion, and finally, Section 4 presents our conclusions.

## 2 Materials and methods

Our data is the result of a food price crowdsourcing initiative implemented in Nigeria between September 2018 and June 2019 (the Food Price Crowdsourcing in Africa (FPCA) initiative) by the Joint Research Centre of the European Commission in collaboration with the International Institute of Tropical Agriculture and Wageningen University and Research. It aimed to assess crowdsourcing's potential to enhance cost-efficiency and timeliness in generating reliable and geo-referenced food price data, accessible in real time to meet the needs of different types of data users, such as governments, private institutions, and value-chain actors (Solano-Hermosilla et al., 2020).

**Fig. 1:**
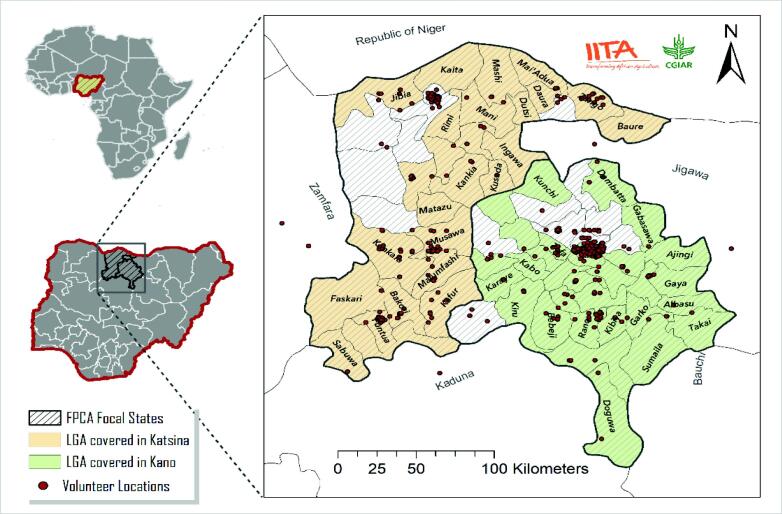
Map showing the focal states of the FPCA project, Local government areas (LGAs) where handbills were distributed for publicity, and the georeferenced locations of the volunteers of the crowd as of the registration time. .

Source: Solano-Hermosilla et al., 2020

The crowdsourcing exercise was carried out in Kano and Katsina States, in the Northern region of Nigeria (Fig. 1). During the first eight weeks of the initiative a media campaign was launched to raise awareness of the initiative and to encourage the registration of prospective volunteers to constitute the crowd. This media campaign included the distribution of leaflets, a radio advertisement, a webpage and word of mouth. The requirements for registration of crowd volunteers included ownership of a smartphone with GPS functionality and the ability to follow openly accessible online instructions. Due to cost constraints, the FPCA did not involve any personal contact between the implementing organisations and the registered volunteers, so no training was provided and efforts were made to create a system that was as simple and user-friendly as possible. The system was built based on Open Data kit (ODK) and deployed on a compatible cloud-based server, ONA, which stores data submissions in real time. Registered volunteers were invited to submit data on prices for food commodities^1^ at different points in the value chain^2^.

The initiative included a reward system designed to be “non-committal”, and yet “promising” for crowd participants. Crowd members submitting prices were eligible to receive a reward of 4 € per submission^3^. This reward was given to a maximum of 30 individuals per day on a “first-submit, first-rewarded” basis, and procedures were in place to make sure that no contributor was rewarded more than once per day, twice per week, or four times per month. Rewards were distributed at the end of each week. Participants were aware that there were a limited number of daily rewards but not of the value of the daily threshold, nor when it was exceeded, to avoid discouraging data submission. Data quality was ensured through the automatic recording of the geolocation of the data entry place and automated data checks through a series of algorithms to extract, clean, and validate price data before upload to the data aggregation platform. Then, based on the auto-recorded time and geo-location, the price was compared to others submitted during the same week within a 12 km radius. Multiple close price points (in time and space) are expected to be similar, leveraging the so-called 'wisdom of the crowd' (Surowiecki, 2004), which allows to filter out spurious data points and confine price values to reasonable ranges. Data submissions exceeding two times the interquartile range were discarded when producing aggregates as they are considered low-quality submissions; however, they are taken into account in our analysis on number of submitted prices. As a result, a validated dataset of food prices was produced on a daily basis and disseminated through a web dashboard. A more detailed description of the methodological approaches can be found in (Solano-Hermosilla et al., 2020).

A total of 737 individuals registered to submit prices, with an average of 16% of them submitting prices in any given week. Descriptive statistics show that the crowd was mainly composed of males (85%), the modal age range was 25–35 years (64%), and nearly all had attained secondary school education or higher. Breakdown of the crowd by occupation shows that the majority of the volunteers either were students (40%) or directly engaged in the agricultural sector (36%). In general, 9 out of 10 volunteers had more than four years’ experience with smartphones. As far as crowd motivation, approximately 93% of the volunteers indicated at registration that they were interested in the data, while a smaller percentage (∼40%) indicated that they were motivated by the possibility of obtaining monetary rewards.

The crowd generated over 18,000 individual submissions, including approximately 170,000 individual price observations or data points. The average submission included nine prices for seven products^4^ from a single point in the value chain. Descriptive statistics of the number of prices submitted per product both at aggregate and individual level are presented in Table SM1 and Table SM2 of the supplementary material.

**Fig. 2:**
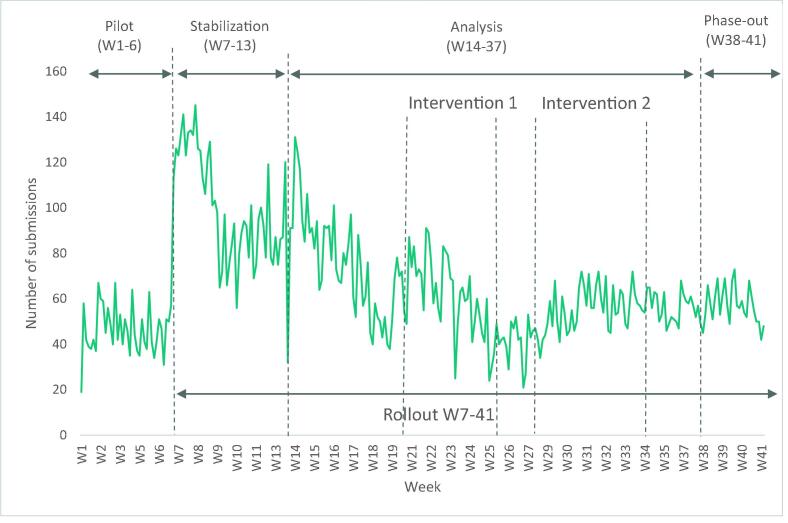
Number of daily records submitted by the crowd over the different project phases between Sep 2018 and Jun 2019. Source: FPCA project on ONA platform.

The 41-week duration of the data collection is divided into two main phases: a pilot covering the first six weeks and the rollout covering the remaining 35 weeks. Within the rollout we distinguish three periods: seven weeks (weeks seven to 13) in which the platform was allowed to stabilize, 24 weeks (weeks 14 to 37) where we focus our analysis and 4 weeks (weeks 38 to 41) as a phase out period (Fig. 2). During the pilot, only a random sample of 200 members of the crowd could submit prices to test the functioning of the system and improve some of its features. During this period, an average of 46 daily submissions were recorded. From week seven onwards, all members were allowed to submit data, and we received an average of 68 daily data records, each containing an average of nine prices. The weekly submission pattern shows a clear difference between weekdays and weekends, and the maximum number of data records were submitted in Weeks seven (141), eight (145) and 14 (131) (Fig. 2).

The data was sourced in three ways. Data on the demographic characteristics of individuals and their households was obtained from the registration questionnaire where information was also provided on awareness of the initiative, motivations for participating, experience with the use of mobile phones and their preferred means for exchanging information. These are time-invariant sample characteristics in our analysis.

Second, data was obtained from submitted prices, such as geo-location, commodity, quality grade, price, unit to which the price refers, market type and distance travelled to market. Only complete submissions^5^ are considered in our analysis. Finally, data was also collected on whether the submission was rewarded or not.

**Fig. 3:**
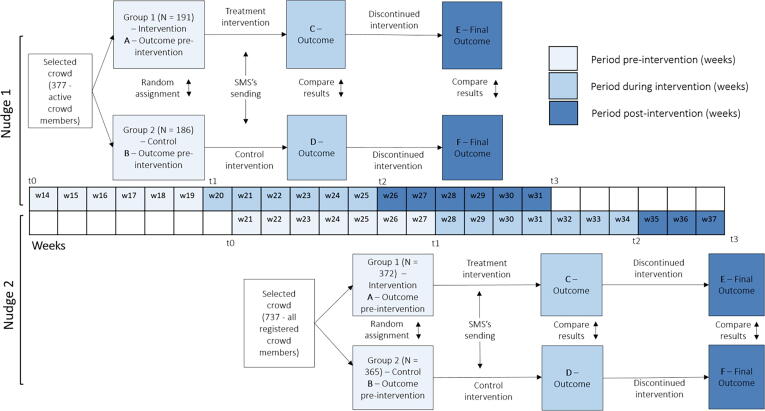
Experimental design of the implementation of the nudges. Source: Own elaboration.

From Week 14 onwards, submissions began to decline and the authors decided to test whether insights from behavioural science could be used to maintain the crowd’s level of submissions. Two different nudges were tested, one based on communicating ‘social norms’ (Intervention 1) and one based on disclosing aggregated crowdsourced information (Intervention 2), both using SMS messages^6^. These nudges were implemented while the financial reward system was still in place following the experimental research design described in Fig. 3.

### 2.1 Intervention 1 – social norm-based nudge

At the end of Week 19, volunteers^7^ were randomly assigned to one of two groups and were kept in this group for the six weeks. For this intervention, randomization only applied to the 377 crowd members who had submitted data at any point. Non-active crowd members (i.e. the remaining 360) were excluded as this nudge used information on the number of submissions. Each Monday, individuals who had submitted prices the week before received an SMS. The control group received an SMS thanking them for participating in the data submission with the following wording:

“Thank you for submitting prices last week to FPCA!”^8^

This allows us to be able to disentangle the effects of receiving some communication from the social norm included in the treatment SMS, thus de facto implementing a random placebo controlled field trial.

The treated group received additional information relating to the number of their price submissions the previous week, the average number of prices submitted by the crowd during the same period, and their percentile ranking in the crowd. This SMS had five alternative versions, depending on the individual’s percentile ranking each week as shown in Table 1.

**Table 1:** Wordings of the SMS’ sent to the treated sub-sample.

Percentile ranking	SMS text [in bold the text submitted to the control group]
[1–10]	***Thank you for submitting****XX****prices last week to FPCA****! On average, participants submitted YY prices, that puts you among the top 10% of participants*
[11–25]	***Thank you for submitting****XX****prices last week to FPCA****! On average, participants submitted YY prices, at least 10% of them submitted more prices than you*
[26–50]	***Thank you for submitting****XX****prices last week to FPCA****! On average, participants submitted YY prices, at least 25% of them submitted more prices than you*
[51–75]	***Thank you for submitting****XX****prices last week to FPCA****! On average, participants submitted YY prices, at least 50% of them submitted more prices than you*
[76–100]	***Thank you for submitting****XX****prices last week to FPCA****! On average, participants submitted YY prices. You are at the lower end, with 75% submitting more than you*

Source: own elaboration

The intervention was discontinued after six weeks. While the experiment was not pre-registered, the basic hypothesis regarding the nudge’s impact on submissions were the following:

H1: Providing information on social norms does not increase submissions during the intervention period.

H2: The impact of the social norm does not persist once the intervention is discontinued.

In order to test these hypotheses, we ran econometric regressions for different temporal settings as described in Table 2.

**Table 2:** Overview of hypotheses testing Nudge 1**.**

Hypothesis	T0 (weeks)	T1 (weeks)	
H1	14–19	20–25	We test H_0_ β_time#treated_ = 0 in Table 7
H2	14–19	26–31	

Note: week numbers correspond to the crowdsourcing project weeks.

### 2.2 Intervention 2 – Information disclosure-based nudge

Following a two-week period without any active communication with the crowd, we implemented a second nudge based on the disclosure of the collectively crowdsourced dataset during seven weeks. All 737 registered volunteers were randomly assigned to one of the two groups, independently from their first assignment. This included active contributors (those who had submitted data at any time during the six weeks prior to the intervention) and non-active contributors (those who had not submitted data during the six weeks prior to the intervention). We included all volunteers with the expectation that the nudge could help mobilise volunteers that did not contribute during the pre-intervention period

The intervention was designed as a weekly SMS text to each of the registered contributors. Similar to Nudge 1, half of the sample received an SMS either thanking them for their contributions (active volunteers) or reminding them that despite being registered they had not contributed (non-active volunteers). The wording of the SMS can be found in Table 3 and depends on the status of the contributor. The other half of the sample received the same message but with the addition of a web link providing access to a web dashboard with several indicators of prices over space and time. The intervention lasted for seven weeks, and the platform remained active for an additional seven weeks after this point.

**Table 3:** Wordings of the SMS sent to the control and treated sub-samples.

Active volunteer	SMS text to the control group	Additional SMS text to the treated group
Yes	*Thank you for your participation in the FPCA project.*	*Explore what you can do with the contributions here:**https://datam.jrc.ec.europa.eu/datam/mashup/FP_NGA**.*
No	*We noticed that you registered in FPCA project, but have not been recently active*	

The idea behind this nudge was to understand whether contributing to the co-creation of public goods (such as a comprehensive market price database) would enhance citizens’ participation through intrinsic motivational factors, such as social contribution, altruism, trust and fun. The basic hypothesis regarding the nudge’s impact on submissions are the following:

H3: Providing access to information does not increase submissions during the intervention period.

H4: The impact of access to information does not persist after the intervention period.

In order to test these hypotheses, we ran econometric regressions using the Difference-in-Difference (DID) approach for different temporal settings as described in Table 4.

**Table 4:** Overview hypotheses testing of Nudge 2.

Hypothesis	T0 (weeks)	T1 (weeks)	
H3	21–27	28–34	We test H_0_ β_time#treated_ = 0 in Table 11
H4	21–27	35–37	

### 2.3 Experimental design

The nudges were implemented following an experimental research design (Fig. 3). The registered crowd was randomly split into two groups in two moments in time. Both random splits were independent from each other. In both cases, one of the groups received the nudge (or treatment), whereas the other half did not. The experimental design permits an analysis based on the gold standard of intervention evaluation: the RCT.

DID models are used to measure the differences between the treated and control groups in terms of the change in an outcome variable (in our case, the number of price submissions) over time (Lechner, 2011). In particular, we aim to estimate two type of impacts: (a) short-term impacts - comparing the change in the average number of price submissions before and during the intervention ([C-A] vs [D-B] in Fig. 3), and (b) long-term impacts: comparing the change in the average number of submissions before and after the intervention ([E-A] vs [F-B]).

Each Monday, during the intervention period, the crowd members received an SMS, which differed between the treatment and control group as described above. We used the DID approach to test for the impact of the nudge on the differences in the number of prices submitted by the treated versus the control group in the implemented RCTs.

We specified linear regression models in which the units of observation and outcome variable ($Y_{i}$) were the number of prices submitted per volunteer (i) in each week (t), thus, over time we have repeated measures for the same volunteers as follows:

$$Y_{\mathrm{it}}=\alpha +\beta Time+\gamma {\mathrm{Treated}}_{i}+\delta \left(Time*{\mathrm{Treated}}_{i}\right)+\theta_{k}X_{k,i,t}+\varphi_{p}Q_{p,i}+\varepsilon_{i}$$

The DID term $\left(Time*{\mathrm{Treated}}_{i}\right)$ was the variable of interest, capturing the difference in changes in the outcome variable between the treated and control groups. Time is a variable that takes a value of 0 if the period is pre-intervention and 1 after. Treated takes a value of 0 if the volunteer belongs to the control group, and 1 if the volunteer belongs to the treated group. The δ coefficient for the DID term estimates the effect of the treatment on the outcome variable. We include a vector of k time variant covariates ($X_{k,i,t})$ and one of p time-invariant ones ($Q_{p,i}$) which are not influenced by the treatment. While time-invariant covariates are exogenous and its effect should be controlled by randomisation, special attention must be given to time-variant covariates to avoid those that may be influenced by the treatment. In our study it is crucial to control for the effect of the individual having received a financial reward or not, and for having received a nudge, in the previous period. Last, $\varepsilon_{i}$ is the error term accounting for unobserved random factors. The analyses were performed as Ordinary Least Square (OLS) regressions, clustering the standard errors per volunteer to deal with the correlation over time of the same volunteers’ observations (Angrist & Pischke, 2008).

An important issue with RCT is avoiding interactions between individuals in the treated and control groups, to prevent spillover effects. While we cannot test for this, in the case of the social norm-based nudge the SMS messages rank individual behaviours and thus have an individual character and should not affect other participants any more than a thank you message. For the second nudge, we were able to test whether the control group accessed the information, which was not the case.

## 3 Results and discussion

### 3.1 Intervention 1 – social norms

We randomly assigned into the treatment and control groups all 377 volunteers who had submitted prices up to week 19. However, while 284 submitted prices at any time during the period of analysis (pre-intervention, intervention and post-intervention), only 168 submitted prices during the intervention period and therefore received one of the two SMS versions used to implement the treatment. The random assignment of volunteers resulted in equivalent samples irrespective of the group analysed (Tables SM3, SM4 and SM5 in the supplementary material). The analysis of the 284 and 168 volunteers estimate the intent to treat (ITT) and the treatment on the treated (TOT), respectively. Due to the nature of the intervention, i.e. SMSs being only sent when individuals reported prices, the pre-intervention, intervention, and post-intervention periods are individual specific. For the analysis, we take into consideration this characteristic of the data. Specifically, the duration and week numbers of the pre-intervention (from week 14 until the week previous to the first SMS received by the individual) and intervention (weeks in which the individual received an SMS) periods are individual specific; and the week numbers for the post-intervention period too but with a common duration for all (six weeks after the last week in which the individual received an SMS). The variables used in the analysis include two outcome variables (number of prices submitted by a volunteer in a given week and percentage of valid prices submitted by a volunteer in a given week) and a set of ‘dummy’ variables to control for time-varying and invariant factors. In particular, as the post-intervention period also covered the second intervention, we controlled for their allocation to the control or treatment group during the second intervention. In the second model reported in Table 7 we also include participants who received a reward for their submissions in the previous week. Financial rewards are extrinsic motivations and should increase participation; not including the variable in the analysis could mask the impact of the nudge. The outcome variable Number of prices submitted is not statistically different between the treated and control group in the pre-treatment period, while for the rest of the covariates used in the analysis, only three presented significant differences (Table 5).

**Table 5:** Description of the dependent, time variant and time invariant variables, and results of t-tests of equality of means at the baseline (pre-treatment) period for the treated and control sub-samples for the nudge, based on social norm (N = 168).

Variables	**Description**	**Mean Control**	**Mean Treated**	**Diff**	**t value**	**pvalue**
Number of prices submitted	The number of prices submitted by a volunteer weekly	25.11	27.04	−1.93	−1.1	0.27
Percentage of valid prices submitted	The percentage of weekly valid prices on total prices submitted by a volunteer	90.39	88.34	2.04	1.7	0.09*
Reward previous week	=1 if the volunteer had been rewarded in the current week, otherwise 0	0.31	0.35	-0.04	−1.15	0.26
Nudge2	=1 if the volunteers is in the treated group in the second intervention	0.47	0.54	-0.06	−1.6	0.11
Gender	=1 if the volunteers is male	0.82	0.9	-0.08	−3	0.00***
Age	=1 if the volunteer is < 30 years old	0.71	0.69	0.02	0.55	0.57
Education	=1 if the volunteers has tertiary education	0.87	0.86	0.01	0.55	0.59
Preferred communication method	=1 if the volunteers prefers SMS	0.68	0.74	-0.06	−1.55	0.12
Farmer	=1 if the volunteer is a farmer	0.54	0.53	0.01	0.35	0.72
Consumer	=1 if the volunteer is a final consumer	0.26	0.28	-0.01	-0.35	0.71
Trader	=1 if the volunteer is a trader	0.09	0.08	0.01	0.25	0.80
Motivation to participate personal	=1 if yes	0.46	0.41	0.05	1.4	0.16
Motivation to participate reward	=1 if yes	0.33	0.43	-0.11	−2.85	0.00***
Motivation to participate data	=1 if yes	0.87	0.95	-0.08	−3.45	0.00***

*** p < 0.01; ** p < 0.05; * p < 0.1.

Table 6 presents descriptive statistics for the average number of price submissions per volunteer in a given week for the treated and control groups in the three periods of analysis (pre-intervention, intervention and post-intervention), which signals towards a bigger increase in submissions for the treated group.

**Table 6:** Average number of price submissions by volunteers in a given week before, during and after the intervention based on the communication of the social norm (nudge 1) – standard errors in parentheses.

	Pre-intervention	Intervention	Post-intervention
All	26.21 (0.87)	31.03 (1.11)	30.18 (1.23)
Treated	27.04 (1.25)	34.89 (1.54)	34.29 (1.77)
Control	25.11 (1.17)	26.74 (1.57)	25.08 (1.62)

**Table 7:** The effects of social norms on the number of weekly price submissions by volunteers, comparing pre-intervention with (a) intervention period and (b) post-treatment period.

	(a) Pre-intervention vs. intervention			(b) Pre-intervention vs. post-intervention		
VARIABLES	Model 1	Model 2	Model 3	Model 1	Model 2	Model 3
time#treated (DID)	6.21*	6.40*	7.28**	7.28	8.08*	8.78*
	(3.53)	(3.50)	(3.61)	(4.90)	(4.78)	(4.81)
time	1.63	0.058	−0.35	−0.029	−2.16	−1.40
	(2.41)	(2.32)	(2.49)	(3.09)	(2.94)	(3.20)
treated	1.93	1.53	0.60	1.93	1.33	1.21
	(3.31)	(3.14)	(3.22)	(3.31)	(3.07)	(3.29)
Reward (previous week)		9.77***	9.46***		14.6***	15.1***
		(1.54)	(1.59)		(2.25)	(2.48)
Nudge2 (=1, treated)						−0.76
						(3.61)
Gender (=1, male)			2.70			−3.23
			(5.36)			(5.45)
Age (=1 < 30 years)			−0.089			2.89
			(3.58)			(4.08)
Education (=1, tertiary education)			3.22			5.88
			(4.63)			(4.85)
Preferred communication method (=1, SMS)			8.41**			12.0***
			(3.32)			(3.68)
Farmer			−3.31			1.97
			(5.13)			(5.63)
Consumer			1.00			2.09
			(5.48)			(5.59)
Trader			−1.55			4.93
			(8.64)			(10.2)
Motivation to participate personal (=1, yes)			−1.26			−2.30
			(3.48)			(4.29)
Motivation to participate reward (=1, yes)			0.97			−2.13
			(3.68)			(4.38)
Motivation to participate data (=1, yes)			7.49***			7.03**
			(2.40)			(2.77)
Constant	25.1***	22.1***	6.64	25.1***	20.6***	1.02
	(2.06)	(1.93)	(9.56)	(2.06)	(1.95)	(12.6)
Observations	1,283	1,283	1,180	1,368	1,368	1,250
R-squared	0.022	0.058	0.098	0.019	0.083	0.134

Results from OLS regressions, robust clustered standard errors at the volunteer ID level in parentheses.

*** p < 0.01, ** p < 0.05, * p < 0.1.

**Fig. 4:**
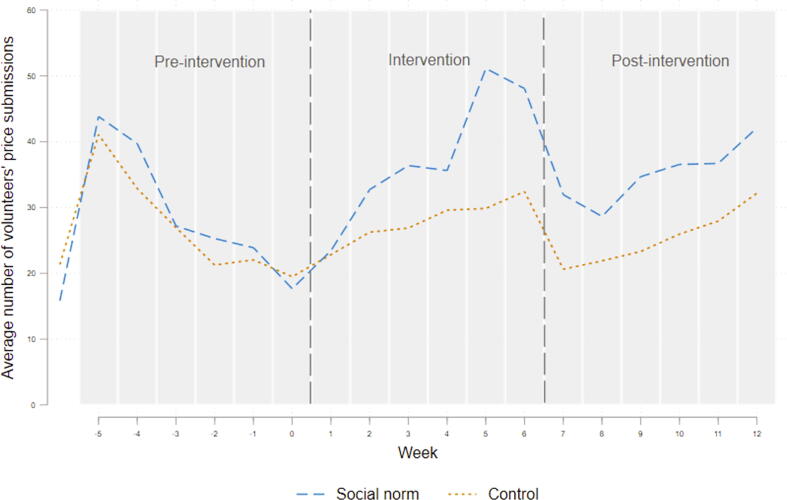
Average number of submissions in a given week by the nudge (nudge 1) and control groups during the pre-intervention, intervention and post-intervention periods.

Both the treatment and control groups followed similar trends in weekly price submissions in the pre-treatment period (Fig. 4). After initiation of the intervention, the number of prices submitted by the volunteers in a given week increased for the treated group and the control group (probably because of receiving an SMS), albeit at a lower rate for the latter.

Table 7 presents the effects of the nudge resulting from the RCT and the applied DID analysis. Panel (a) shows the results of the comparison between the pre-intervention and the intervention periods, while Panel (b) presents the results of the comparison between pre-intervention and post-intervention ones. For each comparison, we ran three models. Model 1 estimated the DID effect, without controlling for any exogenous variable. Model 2 introduced information on whether the volunteer received a monetary reward the week of the submission, to disentangle the effect of the nudge from the effect of the reward. Finally, Model 3 included time-invariant controls capturing whether they have an impact on the submission trends, independently of the group. When reading these controls, a positive and significant value in both groups signals that volunteers with × characteristic (i.e. age < 30) made more submissions than those without it (i.e. age > 30). DID estimates are statistically significant for the intervention and post-intervention comparison and reveals that the social norm generated additional seven price submissions per week during the intervention period and nine during the post-intervention one. The type of message received during the second intervention did not increase the number of submissions. However, rewarded volunteers increased their number of submissions, suggesting that the effect of the financial reward, and that of the social norm nudge, are independent.

These results reject both H1 (i.e. the nudge is not effective during the intervention period) and H2 (i.e. the nudge is not effective after the intervention period). As mentioned above, the analysis of the 168 volunteers is a measure of TOT, if we want to estimate the ITT effect the analysis has to focus on the 377 volunteers. For this sample, we also reject the null hypothesis for the intervention and post-intervention periods (Table SM8).

Once the data was analysed for the main hypotheses, we undertook additional exploratory tests to better understand the impact of the nudge. First, we looked at the type of behaviour activated by the social norm. Following Rogers & Feller (2018), the message providing a descriptive norm can have a dual impact. For those above the descriptive norm it can reinforce the behaviour signalled by the norm. For those below, if the descriptive social norm seems unattainable, it might further discourage the desired behaviour. Here we test whether the nudge works for those already reporting above the average (reinforcing good practice) or below (activating a catching up behaviour). To do this we create two sub-samples based on the contributions submitted during the pre-intervention period^9^. The above (below) average subsample includes both treated and un-treated individuals that reported a number of prices above (below) the average.

Table 8 shows that the impact is only significant for participants who submitted below the crowd’s average Therefore, we observe that descriptive social norms seem to have a positive effect by motivating lagging participants to ‘catch-up’.

**Table 8:** The effects of social norms on the number of weekly price submissions by volunteers, comparing pre-intervention with (a) intervention period and (b) post-treatment period for the sub-groups of volunteers that submitted above and below average at the baseline period in terms of the number of price submissions.

		**(a) Pre-intervention vs. intervention**			**(b) Pre-intervention vs. post-intervention**		
	**Variables**	**Model 1**	**Model 2**	**Model 3**	**Model 1**	**Model 2**	**Model 3**
Above average	time#treated (DID)	−3.38	−2.80	−2.55	4.02	5.19	5.48
		(7.64)	(7.63)	(8.36)	(13.3)	(13.2)	(14.3)
	Observations	453	453	435	456	456	435
	R-squared	0.021	0.034	0.088	0.046	0.070	0.146
Below average	time#treated (DID)	7.91**	7.99**	6.98*	6.07	6.42	6.88*
		(3.30)	(3.28)	(3.60)	(3.99)	(3.89)	(3.72)
	Observations	655	655	589	711	711	634
	R-squared	0.068	0.086	0.110	0.053	0.068	0.136

Results from OLS regressions, robust clustered standard errors at the volunteer ID level in parentheses.

*** p < 0.01, ** p < 0.05, * p < 0.1.

We also checked whether the nudge had an impact on the quality of the prices submitted, but failed to see any significant difference between the treated and control group (Table SM9 to Table SM11 and Fig. SM2 in supplementary materials).

### 3.2 Intervention 2 – Feedback on daily prices (information disclosure)

Following the random allocation for the second nudge, volunteer socio-demographics were significantly different between the intervention and control groups for age (see Table SM12). However, the potential bias due to differences in sample characteristics is addressed by including ‘dummy’ variables to control for both time-varying and time-invariant factors. In particular, we account for the impact of receiving a reward for data submissions in the previous week (as financial rewards can affect trends in participation) and for the type of message received in the first intervention (social norm). As in the previous analyses, we included three attitudinal variables which could affect the utility obtained from the provision of information. These indicated whether the volunteers’ declared their participation was motivated by personal interest, reward, or interest in the data. We considered, in the context of this nudge, that this might have an impact on the number of submissions for volunteers whose participation was motivated by the interest in the collective data. Last, we included a variable to distinguish volunteers reporting commodity prices paid for or obtained in a transaction from those observing the market.

The outcome variables used to analyse the impact of this second nudge consider both the quantity (number) and quality (percentage of valid) of weekly prices submitted (Table 9).

**Table 9:** Description of the variables and results of t-tests of equality of means at the baseline (pre-intervention) period for the treated and control sub-samples for the nudge based on disclosing the collectively produced set of prices.

Variables	Description	Mean Control	Mean Treated	Diff	t value	p value
Number of prices submitted	The number of weekly prices submitted by a volunteer	29.47	28.78	0.69	0.35	0.73
Percentage of valid prices submitted	The percentage of weekly valid prices on total prices submitted by a volunteer	90.89	90.75	0.14	0.15	0.89
Reward (previous week)	=1 if the volunteer had been rewarded in the current week, otherwise 0	0.43	0.40	0.03	1.00	0.32
Nudge 1	=1 if the volunteers is in the treated group in the second intervention	0.51	0.51	0.01	0.25	0.82
Gender	=1 if the volunteers is male	0.95	0.89	0.07	3.55	0.00***
Age	=1 if the volunteer is < 30 years old	0.66	0.67	-0.01	-0.25	0.82
Education	=1 if the volunteers has tertiary education	0.88	0.89	-0.02	-0.85	0.39
Preferred communication method	=1 if the volunteers prefers SMS	0.62	0.64	-0.02	-0.7	0.47
Farmer	=1 if the volunteer is a farmer	0.61	0.51	0.1	2.85	0.00***
Consumer	=1 if the volunteer is a final consumer	0.25	0.22	0.03	0.9	0.38
Trader	=1 if the volunteer is a trader	0.05	0.13	-0.09	−4.4	0.00***
Motivation to participate personal	=1 if yes	0.46	0.41	0.05	1.5	0.13
Motivation to participate reward	=1 if yes	0.38	0.34	0.04	1.15	0.25
Motivation to participate data (=1, yes)	=1 if yes	0.96	0.92	0.04	2	0.05*

*** p < 0.01; ** p < 0.05; * p < 0.1

Table 10 presents the average number of price submissions per volunteer in a given week for the treated and control groups in the three periods of analysis (pre-intervention, intervention and post-intervention), which, contrary to expectations, signals a bigger increase in submissions for the control group.

**Table 10:** Average number of price submissions by volunteers in a given week before (weeks 14–19), during (weeks 20–25) and after (weeks 26–31) the intervention based on the communication of the social norm (nudge 1). Standard deviations in parentheses.

	Pre-intervention	Intervention	Post-intervention
All	29.11 (1.00)	33.87 (1.36)	35.87 (1.97)
Treated	28.78 (1.42)	31.44 (1.90)	35.16 (3.01)
Control	29.47 (1.41)	36.26 (1.95)	36.53 (2.58)

**Fig. 5:**
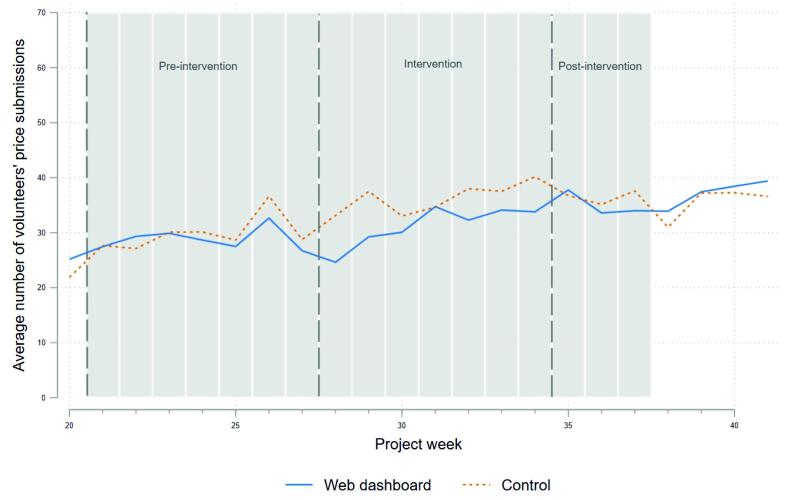
Average number of volunteers’ weekly submissions within the nudge (nudge 2) and control groups.

Focusing on the trends (Fig. 5), the weekly submissions by individuals were almost identical in the pre-intervention period. Surprisingly, during the intervention period, although both groups showed increasing submissions, the control group’s submissions are higher than the treated one throughout the intervention period.

Based on the DID results (Table 11), we fail to reject H3 and H4. The non-significance of the DID coefficient shows that the increases in submissions of both the control and the treated group are equivalent. The positive impact of the financial reward remains unaffected (models 2 and 3), so at least this nudge does not affect the extrinsic motivation generated by the reward. While we cannot directly test it, the lack of impact can be attributed to multiple factors. First, it is possible that the individuals did not understand how to use the tool and the price information, did not find the information provided useful, or had issues with technology (e.g. low-range smartphones, incompatible internet browsers) and connectivity (bad quality internet connection) preventing them from using the tool^10^. In addition, there could have been ‘free-riding’ effects whereby participants think they can benefit from the information tool without contributing to it.

**Table 11:** The effects of price disclosure on the number of weekly price submissions by volunteers, comparing pre-intervention (Weeks 21–27) with (a) intervention period (Weeks 28–34) and (b) post-intervention period (Weeks 35–37).

	a) Pre-intervention vs intervention			b) Pre-intervention vs post-intervention		
VARIABLES	Model 1	Model 2	Model 3	Model 1	Model 2	Model 3
Time#treated (DID)	−4.13	−3.37	−1.04	−0.68	−1.05	−2.13
	(3.64)	(3.59)	(4.00)	(3.69)	(3.70)	(3.99)
Time	6.79**	5.32**	3.73	7.07***	6.17**	7.52***
	(2.78)	(2.59)	(2.51)	(2.50)	(2.48)	(2.39)
Treated	−0.69	−0.11	0.39	−0.69	−0.24	0.54
	(4.29)	(4.03)	(3.87)	(4.29)	(4.10)	(3.98)
Reward (previous week)		16.7***	16.7***		13.0***	12.8***
		(2.69)	(2.82)		(2.69)	(2.90)
Nudge1			11.4**			8.85*
			(5.08)			(4.80)
Gender (=1, male)			2.83			6.72
			(6.80)			(7.70)
Age (=1, <30)			4.51			5.23
			(4.87)			(4.72)
Education (=1, tertiary educ)			5.08			5.86
			(6.27)			(6.23)
Preferred communication method (=1, SMS)			16.0***			14.2***
			(4.28)			(4.15)
Farmer			11.2*			4.71
			(6.64)			(6.58)
Consumer			11.3*			8.90
			(6.56)			(6.71)
Trader			22.1			16.0
			(15.9)			(14.6)
Motivation to participate personal (=1, yes)			0.20			1.14
			(5.64)			(5.01)
Motivation to participate reward (=1, yes)			−3.98			−0.83
			(5.85)			(5.30)
Motivation to participate data (=1, yes)			9.23*			11.0**
			(5.36)			(4.90)
Constant	29.5***	22.2***	–22.9	29.5***	23.8***	−21.9
	(2.92)	(2.54)	(17.4)	(2.92)	(2.79)	(16.2)
Observations	1,527	1,527	1,336	1,120	1,120	975
R-squared	0.008	0.073	0.177	0.010	0.054	0.157

OLS regression, robust clustered standard errors at the volunteer ID level in parentheses.

*** p < 0.01, ** p < 0.05, * p < 0.1.

As for the first nudge, once the data was analysed for the main hypothesis, we undertook some additional exploratory tests to better understand the (lack of) impact of the nudge. The design of this intervention allows us to test whether impacts differ between active and non-active participants, defined as those having submitted prices or not in the six weeks before the intervention. We tested whether the random allocation also holds for these two sub-groups of volunteers and confirm this (see Table SM13 in the supplementary material where only one variable is different for the control and treatment groups for each of the sub-groups). Carrying out the same analysis for both sub-groups separately (Table SM14 in the supplementary material), we see that the DID coefficient is not significant for both; thus, the nudge is ineffective in increasing submissions (activating a behavioural pathway) and increasing participation rates (activating a selection pathway). Second, we checked whether the impact of the information disclosure nudge differs by the role crowd members play in the market. For this, our exploratory hypothesis is that market actors (i.e. those reporting a price they have actually received or paid in a transaction) could be affected differently than those just reporting prices they see in the market place. For the former, the nudge could also activate extrinsic motivations as they can use this information for a self-utility purpose, while the latter would only be driven by intrinsic motivational factors related to contributions to the public good.

Therefore, we split the sample between market actors and observers and ran the same analysis (see Table SM15 in supplementary material). The DID terms are not significant for both type of individuals, thus it seems that information disclosure does not activate extrinsic factors either. Then, we tested whether crowd volunteers that receive the nudge, are more motivated to provide quality information (free of errors) as they perceive the usefulness of the information and care about it. For this, we repeated the analysis but using as dependent variable the percentage of valid prices submitted by each individual in any given week. Again, we failed to find any evidence that the nudge contributed to an increased quality of submissions, either during the intervention or in the post-intervention period (see Fig. SM3, Table SM16 and Table SM17 in supplementary materials).

## 4 Discussion and conclusions

Crowdsourcing food prices is a promising tool for improved market transparency; it offers statistical offices the potential to gather large amounts of complementary information at a low cost using ICT (e.g. internet, mobile apps and smartphones) and direct citizen contributions. Once adequately checked, these resulting datasets of prices can provide real time price data. Yet, ensuring that individuals submit prices, and continue to do so, is a key challenge and even more so for initiatives that do not offer a financial reward or stop offering them. Using a RCT experimental design we explored the use of behavioural tools to help sustain these crowd contributions. In addition to monetary rewards, we implemented two nudges that tried to activate levers to strengthen intrinsic (e.g. competition, cooperation, fun, and altruism) or extrinsic (e.g. self-utility) motivations based on the social norm and disclosing information. Table 12 summarises the results of the hypotheses tested across both interventions.

**Table 12:** Summary of hypotheses and results.

Intervention	H	Description	Result
Social norm	H1	The sub-sample receiving the social norm will not increase their submissions more than the control during the intervention period	rejected
	H2	The impact of the social norm will not persist after the intervention period	rejected
Information disclosure	H3	The sub-sample having access to information will not increase their submissions more than the control during the intervention period	fail to reject
	H4	The impact of access to information will not persist after the intervention period	fail to reject

Opposite results were found for the two types of nudges studied. The social norm-based nudge was effective for increasing participation both during and after the intervention. Furthermore, consistent with previous field experimental research in crowd motivation (Blaschke et al., 2013), our experiment showed that the effect of the financial reward (extrinsic motivation) and the social norm nudge (intrinsic motivation) are independent. For both nudges we did not find any impact on the percentage of valid prices reported.

The second result shows that information appears not to engage people into submitting more prices. This is in line with a recent *meta*-analysis of behavioural interventions that concludes that social comparison messages are more effective than information-based strategies in motivating people in action on climate change, the latter having little impact on unmotivated individuals (Nisa et al., 2019). Our results also show that the information disclosure nudge does not affect the impact of the financial reward incentive on promoting higher submissions.

Additional exploratory analysis shows that the effect of the social norm nudge is mainly driven by the behaviour of volunteers previously reporting less prices, thus suggesting a catching up behavioural response. The information disclosure-based nudge did not show an effect on increasing the number of contributions, neither during the intervention, nor after it. It seems that the increase in submissions observed during, and after, the intervention is related to the interaction between the platform and the volunteers (i.e. sending an SMS) irrespective of its content. Even when undertaking the analysis separately for “active” and “non-active” contributors, and for “market actors contributors” and “market observers contributors”, results showed no impact. The former discards both a behavioural and a selection story; while the latter supports discarding that information disclosure activated extrinsic factors related to self-utility. This last conclusion should be further tested.

This study provides, to the best of our knowledge, one of the first experimental evidence on the use of behavioural tools in crowdsourcing to sustain crowd motivation. Our findings can be useful in the design of future crowdsourcing initiatives which aim to engage people to report data, or to conduct a task, in similar or other fields. In particular, we recommend including interaction with users in the design of the crowdsourcing platform as participants showed an increase in submissions, irrespective of the content of the SMS they received, in both interventions. Furthermore, nudges based on descriptive norms seem to increase submissions and this increase is due to the reaction of crowd members who are not contributing the most, i.e. those reporting below the average.

However, our study is not without limitations. First, as for all applied RCTs despite the exploratory analysis we undertake we cannot fully identify the causal mechanism underlying the impact and lack of impact behind our two nudges or validity of the findings beyond our case study. For the social norm based nudge, it seems that it triggers a catching-up behaviour for volunteers reporting below average, but this cannot be fully supported. For the information disclosure nudge, it seems that individuals do not value the information or the format in which the information is provided, but again this cannot be proven. The external validity of the interventions needs to be reinforced by additional studies. Second, some characteristics of our application could have been improved. In particular, the nudge is tested with a financial reward system in place. Therefore, even when we see that the impact of nudges and extrinsic financial motivations are independent, we cannot conclude that the nudge alone would have the same effect in the absence of financial rewards.

Last, our results also identify areas for further research to maximize the potential of nudges in supporting crowdsourcing initiatives. First, it would be interesting to see whether nudges have an impact on the quality of submissions, measuring this as a deviation from prices coming from other objective sources. In addition, alternative nudges could be tested to see how to mobilize inactive crowd members and improve the crowd creation process. Specifically, for the nudge based on information disclosure, the focus could lie on user needs and the use of technologies better tailored to the local condition (e.g. a simple SMS with price statistics rather than a web dashboard). And finally, we would suggest investigating whether information provision has an effect on buying and selling behaviour, as evidence on this, at least for improved prices for producers, is inconclusive (Belay and Ayalew, 2020, Fafchamps and Minten, 2012, Hildebrandt et al., 2015, Mitchell, 2017).

## CRediT authorship contribution statement

**Gloria Solano-Hermosilla:** Conceptualization, Methodology, Validation, Formal analysis, Writing – original draft, Writing – review & editing, Visualization, Project administration. **Jesus Barreiro-Hurle:** Conceptualization, Methodology, Validation, Formal analysis, Writing – original draft, Supervision. **Julius B. Adewopo:** Investigation, Visualization, Writing – review & editing. **Celso Gorrín-González:** Software, Data curation, Writing – review & editing.

## Footnotes

1 Prices could be submitted for five products (local rice, imported rice, maize, beans and soybeans)

2 Prices could be reported at three stages of the value chain: farm-gate, wholesale markets and retail markets.

3 For example, 4 and 5 euros per submission were provided by the FPCA (Solano-Hermosilla et al., 2020) and World Bank (Hamadeh et al., 2013) crowdsourcing initiatives in African countries, respectively. Other initiatives provide weekly rewards of between 8 and 11 euros per data collector (e.g. SIMA in Niger and Sonagess in Burkina Faso) (Zeug et al., 2017).

4 Submissions could include prices for different varieties (e.g. quality grades) of the same product.

5 Submissions that have at least one price with GPS location, information on the product and unit to which it refers to and type of market where it was observed.

6 In a survey carried out during the registration of volunteers in the app, the majority indicated that SMS was their preferred communication option.

7 The crowd included any volunteer who had submitted data at any point in time during the first nineteen weeks of the FPCA initiative.

8 The grammatical construction of the SMS text is not perfect, however due to the character limitations associated with this means of communication (160 characters) some concessions to proper grammar had to be made.

9 We thank a reviewer for suggesting this experimental set-up for the analysis.

10 When presenting the results of the project to the local authorities in the capital of Nigeria (where connectivity should be better than in the study areas) one of the authors experienced how difficult it was to access the web dashboard from the available wifi.
