# Supplementary material for: Increasing engagement in price crowdsourcing initiatives: Using nudges in Nigeria
Source: World Dev. 2022 Apr;152:105818. doi: 10.1016/j.worlddev.2022.105818 (PMC8886569; doi:10.1016/j.worlddev.2022.105818)
Supplement: Supplementary data 1 [file mmc1.docx]

# SUPPLEMENTARY MATERIAL FOR SOLANO-HERMOSILLA ET AL (2022)

**Table SM1.** Descriptive statistics (means and percentiles) of the weekly number of prices submitted and the weekly number of valid prices submitted price per commodity (per volunteer) during the period of analysis (week 14 to week 37).

|  | **Price submissions** | | | | | | **Valid price submissions** | | | | | |
| --- | --- | --- | --- | --- | --- | --- | --- | --- | --- | --- | --- | --- |
| **commodity** | **Mean** | **p25** | **Median (p50)** | **p75** | **max** | **min** | **Mean** | **p25** | **Median (p50)** | **p75** | **max** | **min** |
| Local rice | 35 | 11 | 22 | 49 | 248 | 3 | 24 | 8 | 16 | 35 | 127 | 1 |
| Thailand rice | 25 | 8 | 16 | 38 | 185 | 2 | 19 | 5 | 12 | 30 | 117 | 1 |
| Indian rice | 20 | 8 | 12 | 25 | 205 | 2 | 15 | 6 | 10 | 19 | 113 | 1 |
| White maize | 23 | 8 | 15 | 32 | 216 | 2 | 18 | 6 | 12 | 26 | 86 | 1 |
| Yellow maize | 23 | 7 | 12 | 35 | 133 | 3 | 19 | 5 | 9 | 25 | 116 | 1 |
| Red beans | 33 | 10 | 21 | 42 | 233 | 6 | 24 | 8 | 15 | 34 | 104 | 1 |
| White beans | 34 | 11 | 26 | 49 | 243 | 5 | 25 | 8 | 17 | 35 | 117 | 1 |
| Soybean | 22 | 8 | 14 | 31 | 169 | 2 | 17 | 5 | 11 | 25 | 75 | 1 |

**Table SM2.** Descriptive statistics (means and percentiles) of the weekly number of prices submitted per commodity (total submissions) during the period of analysis (week 14 to week 37).

|  | **Price submissions** | | | | | | **Valid price submissions** | | | | | |
| --- | --- | --- | --- | --- | --- | --- | --- | --- | --- | --- | --- | --- |
| **commodity** | **Mean** | **p25** | **Median (p50)** | **p75** | **max** | **min** | **Mean** | **p25** | **Median (p50)** | **p75** | **max** | **min** |
| Local rice | 1,021 | 832 | 981 | 1,231 | 1,474 | 610 | 698 | 575 | 707 | 796 | 1,045 | 400 |
| Thailand rice | 441 | 255 | 481 | 576 | 773 | 110 | 326 | 152 | 350 | 463 | 612 | 100 |
| Indian rice | 203 | 103 | 203 | 301 | 522 | 26 | 141 | 64 | 136 | 188 | 415 | 15 |
| White maize | 414 | 287 | 431 | 531 | 640 | 177 | 313 | 203 | 328 | 393 | 495 | 144 |
| Yellow maize | 365 | 270 | 321 | 428 | 699 | 119 | 283 | 217 | 256 | 343 | 546 | 78 |
| Red beans | 381 | 262 | 364 | 449 | 759 | 99 | 264 | 183 | 248 | 344 | 521 | 63 |
| White beans | 831 | 652 | 840 | 983 | 1,586 | 393 | 590 | 456 | 598 | 725 | 1,101 | 278 |
| Soybean | 407 | 314 | 414 | 540 | 754 | 97 | 295 | 209 | 303 | 398 | 550 | 76 |

**Table SM3.** Description of the characteristics of the treated and control sub-samples for the nudge based on communicating the social norm (N=168 treated volunteers).

|  | **Nudge 1: Social norm** | | | | | | | |
| --- | --- | --- | --- | --- | --- | --- | --- | --- |
|  | **Treated** | | | **Control** | | **Full sample** | | **P-value** |
| **Variable** | **n** | **%** | | **n** | **%** | **n** | **%** |  |
| Total | 92 | 100% | | 76 | 100% | 168 | 100% |  |
| Gender |  |  | |  |  |  |  | 0.24 |
| Female | 9 | 10% | | 12 | 16% | 21 | 13% |  |
| Male | 83 | 90% | | 64 | 84% | 147 | 88% |  |
| Education |  |  | |  |  |  |  | 0.66 |
| Primary | 0 | 0% | | 0 | 0% | 0 | 0% |  |
| Secondary | 13 | 14% | | 9 | 12% | 22 | 13% |  |
| Tertiary | 79 | 86% | | 67 | 88% | 146 | 87% |  |
| Food chain stage |  |  | |  |  |  |  | 0.99 |
| Farmer | 46 | 50% | | 41 | 54% | 87 | 52% |  |
| Final consumer | 25 | 27% | | 19 | 25% | 44 | 26% |  |
| Retailer and wholesaler | 9 | 10% | | 7 | 9% | 16 | 10% |  |
| Other | 12 | 13% | | 9 | 12% | 21 | 13% |  |
| Preferred communication channel |  |  | |  |  |  |  | 0.12 |
| App | 16 | 17% | | 4 | 5% | 20 | 12% |  |
| Email | 11 | 12% | | 13 | 17% | 24 | 14% |  |
| SMS | 57 | 62% | | 51 | 67% | 108 | 64% |  |
| Web | 0 | 0% | | 1 | 1% | 1 | 1% |  |
| Unknown | 8 | 9% | | 7 | 9% | 15 | 9% |  |
| Nudge 2 |  |  | |  |  |  |  | 0.67 |
| Treated | 49 | 53% | | 38 | 50% | 87 | 52% |  |
| Control | 43 | 47% | | 38 | 50% | 81 | 48% |  |
| Personal motivation |  | |  |  |  |  |  | 0.63 |
| Yes | 39 | | 42% | 35 | 46% | 74 | 37% |  |
| No | 53 | | 58% | 41 | 54% | 94 | 63% |  |
| Reward motivation |  | |  |  |  |  |  | 0.47 |
| Yes | 39 | | 42% | 28 | 37% | 67 | 44% |  |
| No | 53 | | 58% | 48 | 63% | 101 | 56% |  |
| Data motivation |  | |  |  |  |  |  | 0.29 |
| Yes | 80 | | 87% | 61 | 80% | 141 | 84% |  |
| No | 4 | | 4% | 8 | 11% | 12 | 7% |  |
| No answer | 8 | | 9% | 7 | 9% | 15 | 9% |  |
|  | **Mean** |  | | **Mean** |  | **Mean** |  |  |
| Age | 28.01 |  | | 28.05 |  | 28.02 |  | 0.96 |
| Avg. years smartphone use | 6.29 |  | | 6.09 |  | 6.20 |  | 0.58 |

Note: p-values are the results of Chi-Square tests of independence of categorical variables, or from t-tests of equality for continuous variables.

**Table SM4.** Description of the characteristics of the treated and control sub-samples for the nudge based on communicating the social norm (N=284, submitted prices at any point during the period of analysis of this nudge―pre-, during and post-intervention).

|  | **Nudge 1: Social norm** | | | | | | |
| --- | --- | --- | --- | --- | --- | --- | --- |
|  | **Treatment** | | **Control** | | **Full sample** | | **P-value** |
| **Variable** | **n** | **%** | **n** | **%** | **n** | **%** |  |
| Total | 148 | 100% | 136 | 100% | 284 | 100% |  |
| Gender |  |  |  |  |  |  | 0.65 |
| Female | 19 | 13% | 20 | 15% | 39 | 14% |  |
| Male | 129 | 87% | 116 | 85% | 245 | 86% |  |
| Education |  |  |  |  |  |  | 0.65 |
| Primary | 0 | 0% | 0 | 0% | 0 | 0% |  |
| Secondary | 17 | 11% | 18 | 13% | 35 | 12% |  |
| Tertiary | 131 | 89% | 118 | 87% | 249 | 88% |  |
| Food chain stage |  |  |  |  |  |  | 0.57 |
| Farmer | 67 | 45% | 64 | 47% | 131 | 46% |  |
| Final consumer | 53 | 36% | 41 | 30% | 94 | 33% |  |
| Retailer and wholesaler | 14 | 9% | 14 | 10% | 28 | 10% |  |
| Other | 14 | 9% | 17 | 13% | 31 | 11% |  |
| Preferred communication channel |  |  |  |  |  |  | 0.20 |
| App | 21 | 14% | 8 | 6% | 29 | 10% |  |
| Email | 23 | 16% | 22 | 16% | 45 | 16% |  |
| SMS | 90 | 61% | 95 | 70% | 185 | 65% |  |
| Web | 5 | 3% | 3 | 2% | 8 | 3% |  |
| Unknown | 12 | 6% | 10 |  | 22 | 6% |  |
| Nudge 2 |  |  |  |  |  |  | 0.93 |
| Treated | 70 | 47% | 65 | 48% | 135 | 48% |  |
| Control | 78 | 53% | 71 | 52% | 149 | 52% |  |
| Age | 27.51 |  | 27.55 |  | 27.53 |  | 0.94 |
| Avg. years smartphone use | 6.3 |  | 6.4 |  | 6.4 |  | 0.98 |

Note: p-values are the results of Chi-Square tests of independence between categorical variables, or from t-tests of equality for continuous variables.

**Table SM5.** Description of the characteristics of the treated and control sub-samples for the nudge based on communicating the social norm (N=377, full sample).

|  | **Nudge 1: Social norm** | | | | | | |
| --- | --- | --- | --- | --- | --- | --- | --- |
|  | **Treated** | | **Control** | | **Full sample** | | **P-value** |
| **Variable** | **n** | **%** | **n** | **%** | **n** | **%** |  |
| Total | 191 | 100% | 186 | 100% | 377 | 100% |  |
| Gender |  |  |  |  |  |  | 0.36 |
| Female | 31 | 16% | 24 | 13% | 55 | 15% |  |
| Male | 160 | 84% | 162 | 87% | 322 | 85% |  |
| Education |  |  |  |  |  |  | 0.41 |
| Primary | 0 | 0% | 1 | 1% | 1 | 0% |  |
| Secondary | 24 | 13% | 29 | 16% | 53 | 14% |  |
| Tertiary | 167 | 87% | 156 | 84% | 323 | 86% |  |
| Food chain stage |  |  |  |  |  |  | 0.41 |
| Farmer | 80 | 42% | 88 | 47% | 168 | 45% |  |
| Final consumer | 73 | 38% | 56 | 30% | 129 | 34% |  |
| Retailer and wholesaler | 19 | 10% | 20 | 11% | 39 | 10% |  |
| Other | 19 | 10% | 22 | 12% | 41 | 11% |  |
| Preferred communication channel |  |  |  |  |  |  | 0.23 |
| App | 24 | 13% | 12 |  | 36 | 10% |  |
| Email | 33 | 17% | 27 |  | 60 | 16% |  |
| SMS | 117 | 61% | 131 |  | 248 | 66% |  |
| Web | 5 | 3% | 6 |  | 11 | 3% |  |
| Unknown | 12 | 6% | 10 |  | 22 | 6% |  |
| Nudge 2 |  |  |  |  |  |  | 0.43 |
| Treated | 91 | 48% | 81 | 44% | 172 | 46% |  |
| Control | 100 | 52% | 105 | 56% | 205 | 54% |  |
|  | **Mean** |  | **Mean** |  | **Mean** |  |  |
|  |  |  |  |  |  |  |  |
| Age | 27.25 |  | 27.46 |  | 27.35 |  | 0.69 |
| Avg. years smartphone use | 6.3 |  | 6.5 |  | 6.4 |  | 0.43 |

Note: p-values are the results of Chi-Square tests of independence between categorical variables, or from t-tests of equality for continuous variables.

**Table SM6.** Description of the dependent (Number of prices submitted and Percentage of valid prices submitted), time variant and time invariant variables, and results of t-tests of equality of means at the baseline (pre-treatment) period for the treated and control sub-samples for the nudge, based on social norm (N=284).

| **Variables** | **Description** | **Mean Control** | **Mean Treated** | **Diff** | **t value** | **pvalue** |
| --- | --- | --- | --- | --- | --- | --- |
| **Number of prices submitted** | The number of prices submitted by a volunteer weekly | 22.27 | 24.61 | -2.34 | -1.65 | .10 |
| **Percentage of valid prices submitted** | The percentage of weekly valid prices on total prices submitted by a volunteer | 89.68 | 87.60 | 2.08 | 1.95 | .05* |
| **Reward previous week** | =1 if the volunteer had been rewarded in the current week, otherwise 0 | .28 | .32 | -.04 | -1.35 | .18 |
| **Nudge2** | =1 if the volunteers is in the treated group in the second intervention | .51 | .54 | -.02 | -.65 | .52 |
| **Gender** | =1 if the volunteers is male | .83 | .87 | -.04 | -1.65 | .10 |
| **Age** | =1 if the volunteer is <30 years old | .73 | .73 | 0 | -.1 | .92 |
| **Education** | =1 if the volunteers has tertiary education | .87 | .86 | .01 | .5 | .62 |
| **Preferred communication method** | =1 if the volunteers prefers SMS | .67 | .7 | -.03 | -.95 | .33 |
| **Farmer** | =1 if the volunteer is a farmer | .48 | .49 | -.01 | -.35 | .73 |
| **Consumer** | =1 if the volunteer is a final consumer | .33 | .33 | 0 | 0 | .98 |
| **Trader** | =1 if the volunteer is a trader | .09 | .09 | 0 | 0 | .99 |
| **Motivation to participate personal** | =1 if yes | .4 | .41 | -.01 | -.3 | .78 |
| **Motivation to participate reward** | =1 if yes | .32 | .4 | -.08 | -2.45 | .02** |
| **Motivation to participate data (=1, yes)** | =1 if yes | .89 | .95 | -.06 | -3.3 | .00*** |

*** p<0.01; ** p<0.05; * p<0.1

**Table SM7**. Average number of price submissions by volunteers in a given week before, during and after the intervention based on the communication of the social norm (nudge 1) – standard errors in parentheses.

|  | Pre-intervention | Intervention | Post-intervention |
| --- | --- | --- | --- |
| All | 23.55  (0.70) | 29.93  (1.07) | 31.97  (1.45) |
| Treated | 24.61  (1.02) | 34.01  (1.51) | 37.92  (2.18) |
| Control | 22.27  (0.91) | 25.56  (1.47) | 25.34  (1.80) |


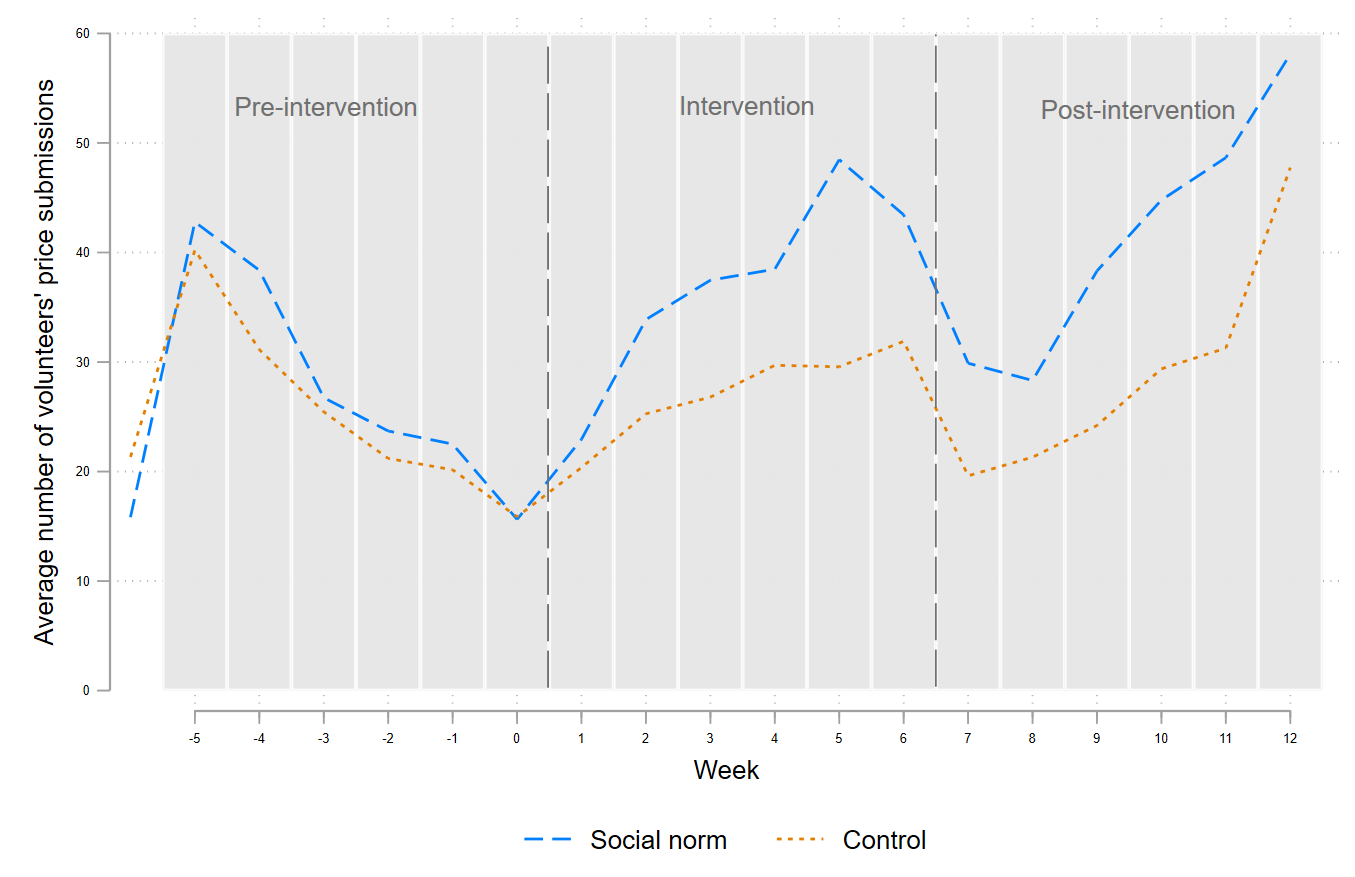


**Figure SM1.** Average number of submissions by volunteers in a given week before, during and after the intervention based on the communication of the social norm (nudge 1) by the nudge and control groups

**Table SM8.** The effects (ITT) of social norms on the number of weekly price submissions by volunteers, comparing pre-intervention with (a) intervention period and (b) post-treatment period.

|  | (a) Pre-intervention vs. intervention | | | (b) Pre-intervention vs. post-intervention | | |
| --- | --- | --- | --- | --- | --- | --- |
| VARIABLES | Model 1 | Model 2 | Model 3 | Model 1 | Model 2 | Model 3 |
|  |  |  |  |  |  |  |
| time#treated (DID) | 6.11* | 6.14* | 6.45* | 10.2* | 11.0** | 12.2** |
|  | (3.34) | (3.30) | (3.46) | (5.67) | (5.51) | (5.63) |
| time | 3.29 | 1.48 | 1.30 | 3.08 | 0.028 | -0.33 |
|  | (2.33) | (2.21) | (2.39) | (3.52) | (3.23) | (3.53) |
| treated | 2.34 | 1.89 | 1.62 | 2.34 | 1.75 | 1.60 |
|  | (2.61) | (2.44) | (2.46) | (2.61) | (2.40) | (2.48) |
| Reward (previous week) |  | 11.1*** | 11.0*** |  | 14.7*** | 15.0*** |
|  |  | (1.45) | (1.47) |  | (2.11) | (2.24) |
| Nudge2 (=1, treated) |  |  |  |  |  | 0.65 |
|  |  |  |  |  |  | (3.20) |
| Gender (=1, male) |  |  | 1.24 |  |  | -3.31 |
|  |  |  | (4.15) |  |  | (4.12) |
| Age (=1 <30 years) |  |  | -1.70 |  |  | 0.088 |
|  |  |  | (3.15) |  |  | (3.73) |
| Education (=1, tertiary education) |  |  | 1.56 |  |  | 1.73 |
|  |  |  | (3.81) |  |  | (4.19) |
| Preferred communication method (=1, SMS) |  |  | 7.98*** |  |  | 9.64*** |
|  |  |  | (2.58) |  |  | (2.93) |
| Farmer |  |  | -2.33 |  |  | 2.88 |
|  |  |  | (4.47) |  |  | (4.86) |
| Consumer |  |  | -0.71 |  |  | 0.30 |
|  |  |  | (4.64) |  |  | (4.58) |
| Trader |  |  | -1.16 |  |  | 4.50 |
|  |  |  | (7.40) |  |  | (8.91) |
| Motivation to participate personal (=1, yes) |  |  | -0.017 |  |  | -1.32 |
|  |  |  | (2.87) |  |  | (3.78) |
| Motivation to participate reward (=1, yes) |  |  | 1.26 |  |  | -0.67 |
|  |  |  | (3.05) |  |  | (3.83) |
| Motivation to participate data (=1, yes) |  |  | 6.28*** |  |  | 7.17*** |
|  |  |  | (2.21) |  |  | (2.48) |
| Constant | 22.3*** | 19.2*** | 7.91 | 22.3*** | 18.2*** | 4.57 |
|  | (1.59) | (1.46) | (8.35) | (1.59) | (1.50) | (11.4) |
|  |  |  |  |  |  |  |
| Observations | 1,554 | 1,554 | 1,448 | 1,472 | 1,472 | 1,371 |
| R-squared | 0.031 | 0.079 | 0.115 | 0.044 | 0.109 | 0.147 |

Results from OLS regressions, robust clustered standard errors at the volunteer ID level in parentheses

*** p<0.01, ** p<0.05, * p<0.1

**Table SM9.** Description of the dependent (Percentage of valid prices submitted), time variant and time invariant variables, and results of t-tests of equality of means at the baseline (pre-treatment) period for the treated and control sub-samples for the nudge, based on social norm (N=168).

| **Variables** | **Description** | **Mean Control** | **Mean Treated** | **Diff** | **t value** | **pvalue** |
| --- | --- | --- | --- | --- | --- | --- |
| **Percentage of valid prices submitted** | The percentage of weekly valid prices on total prices submitted by a volunteer | 90.39 | 88.34 | 2.04 | 1.7 | .09* |
| **Reward (previous week)** | =1 if the volunteer had been rewarded in the current week, otherwise 0 | .31 | .35 | -.04 | -1.15 | .26 |
| **Nudge2** | =1 if the volunteers is in the treated group in the second intervention | .47 | .54 | -.06 | -1.6 | .11 |
| **Gender** | =1 if the volunteers is male | .82 | .9 | -.08 | -3 | .00*** |
| **Age** | =1 if the volunteer is <30 years old | .71 | .69 | .02 | .55 | .57 |
| **Education** | =1 if the volunteers has tertiary education | .87 | .86 | .01 | .55 | .59 |
| **Preferred communication method** | =1 if the volunteers prefers SMS | .68 | .74 | -.06 | -1.55 | .12 |
| **Farmer** | =1 if the volunteer is a farmer | .54 | .53 | .01 | .35 | .72 |
| **Consumer** | =1 if the volunteer is a final consumer | .26 | .28 | -.01 | -.35 | .71 |
| **Trader** | =1 if the volunteer is a trader | .09 | .08 | .01 | .25 | .8 |
| **Motivation to participate personal** | =1 if yes | .46 | .41 | .05 | 1.4 | .16 |
| **Motivation to participate reward** | =1 if yes | .33 | .43 | -.11 | -2.85 | 00*** |
| **Motivation to participate data (=1, yes)** | =1 if yes | .87 | .95 | -.08 | -3.45 | 00*** |

*** p<0.01; ** p<0.05; * p<0.1

**Table SM10.** Average percentage of valid price submissions by volunteers in a given week before, during and after the intervention based on the communication of the social norm (nudge 1) – standard errors in parentheses.

|  | Pre-intervention | Intervention | Post-intervention |
| --- | --- | --- | --- |
| All | 89.22  (0.59) | 90.29  (0.57) | 90.44  (0.53) |
| Treated | 88.34  (0.82) | 89.06  (0.80) | 89.59  (0.70) |
| Control | 90.39  (0.83) | 91.69  (0.82) | 91.49  (0.80) |

**Table SM11.** The effects of social norms on the **percentage of weekly valid** price submissions by volunteers, comparing pre-intervention with (a) intervention period and (b) post-treatment period.

|  | (a) Pre-intervention vs. intervention | | | (b) Pre-intervention vs. post-intervention | | |
| --- | --- | --- | --- | --- | --- | --- |
| VARIABLES | Model 1 | Model 2 | Model 3 | Model 1 | Model 2 | Model 3 |
|  |  |  |  |  |  |  |
| time#treated (DID) | -0.59 | -0.61 | -1.91 | 0.14 | 0.087 | -0.59 |
|  | (1.86) | (1.87) | (1.83) | (1.85) | (1.84) | (1.87) |
| time | 1.31 | 1.45 | 1.91 | 1.10 | 1.26 | 1.04 |
|  | (1.25) | (1.26) | (1.23) | (1.38) | (1.38) | (1.40) |
| treated | -2.04 | -2.01 | -1.63 | -2.04 | -2.00 | -1.42 |
|  | (1.68) | (1.67) | (1.69) | (1.68) | (1.67) | (1.69) |
| Reward (previous week) |  | -0.80 | -1.49 |  | -1.05 | -1.66* |
|  |  | (1.02) | (1.06) |  | (0.91) | (0.93) |
| Nudge2 (=1, treated) |  |  |  |  |  | 0.24 |
|  |  |  |  |  |  | (1.48) |
| Gender (=1, male) |  |  | 0.31 |  |  | 0.29 |
|  |  |  | (1.67) |  |  | (1.61) |
| Age (=1 <30 years) |  |  | -4.40*** |  |  | -2.68 |
|  |  |  | (1.59) |  |  | (1.72) |
| Education (=1, tertiary education) |  |  | -2.43 |  |  | -0.88 |
|  |  |  | (2.23) |  |  | (1.98) |
| Preferred communication method (=1, SMS) |  |  | -3.41** |  |  | -4.22*** |
|  |  |  | (1.50) |  |  | (1.38) |
| Farmer |  |  | 0.94 |  |  | -2.62 |
|  |  |  | (2.60) |  |  | (2.40) |
| Consumer |  |  | 2.93 |  |  | 0.071 |
|  |  |  | (2.78) |  |  | (2.51) |
| Trader |  |  | 1.13 |  |  | -3.66 |
|  |  |  | (2.70) |  |  | (2.72) |
| Motivation to participate personal (=1, yes) |  |  | -0.93 |  |  | -0.46 |
|  |  |  | (1.43) |  |  | (1.39) |
| Motivation to participate reward (=1, yes) |  |  | 2.90* |  |  | 2.34 |
|  |  |  | (1.49) |  |  | (1.45) |
| Motivation to participate data (=1, yes) |  |  | 0.49 |  |  | -1.58 |
|  |  |  | (2.28) |  |  | (1.81) |
| Constant | 90.4*** | 90.6*** | 95.5*** | 90.4*** | 90.7*** | 98.5*** |
|  | (1.03) | (1.09) | (4.98) | (1.03) | (1.10) | (4.57) |
|  |  |  |  |  |  |  |
| Observations | 1,250 | 1,250 | 1,147 | 1,330 | 1,330 | 1,216 |
| R-squared | 0.008 | 0.008 | 0.061 | 0.006 | 0.008 | 0.049 |

Results from OLS regressions, robust clustered standard errors at the volunteer ID level in parentheses

*** p<0.01, ** p<0.05, * p<0.1

**Table SM12.** Description of the characteristics of the treated and control sub-samples for the nudge based on disclosing the collectively produced set of prices.

|  | **Nudge 2: Disclosing collective data** | | | | | | |
| --- | --- | --- | --- | --- | --- | --- | --- |
|  | **Dashboard** | | **Control** | | **Full sample** | | **P-value** |
| **Variable** | **n** | **%** | **n** | **%** | **n** | **%** |  |
| Total | 372 | 100% | 365 | 100% | 737 | 100% |  |
| Gender |  |  |  |  |  |  | 0.36 |
| Female | 61 | 16% | 51 | 14% | 112 | 15% |  |
| Male | 311 | 84% | 314 | 86% | 625 | 85% |  |
| Education |  |  |  |  |  |  | 0.54 |
| Primary | 1 | 0% | 1 | 0% | 2 | 0% |  |
| Secondary | 49 | 13% | 52 | 14% | 101 | 14% |  |
| Tertiary | 322 | 87% | 312 | 85% | 634 | 86% |  |
| Food chain stage |  |  |  |  |  |  | 0.15 |
| Farmer | 151 | 41% | 167 | 46% | 318 | 43% |  |
| Final consumer | 138 | 37% | 119 | 33% | 257 | 35% |  |
| Retailer and wholesaler | 41 | 11% | 46 | 13% | 87 | 12% |  |
| Other | 42 | 11% | 33 | 9% | 75 | 10% |  |
| Preferred communication channel | |  |  |  |  |  | 0.99 |
| App | 31 | 8% | 28 | 8% | 59 | 8% |  |
| Email | 67 | 18% | 70 | 19% | 137 | 19% |  |
| SMS | 236 | 63% | 231 | 63% | 467 | 63% |  |
| Web | 13 | 3% | 11 | 3% | 24 | 3% |  |
| Unknown | 25 | 7% | 25 | 7% | 50 | 7% |  |
| Nudge 1 |  |  |  |  |  |  | 0.07 |
| Treated | 105 | 28% | 81 | 22% | 186 | 25% |  |
| Control | 100 | 27% | 91 | 25% | 191 | 26% |  |
| Non participant | 167 | 45% | 193 | 53% | 360 | 49% |  |
| Personal motivation |  |  |  |  |  |  | 0.57 |
| Yes | 141 | 38% | 131 | 36% | 272 | 37% |  |
| No | 231 | 62% | 234 | 64% | 465 | 63% |  |
| Reward motivation |  |  |  |  |  |  | 0.21 |
| Yes | 139 | 37% | 153 | 42% | 292 | 40% |  |
| No | 233 | 63% | 212 | 58% | 445 | 60% |  |
| Data motivation |  |  |  |  |  |  | 0.40 |
| Yes | 309 | 83% | 313 | 86% | 622 | 84% |  |
| No | 38 | 10% | 27 | 7% | 65 | 9% |  |
| No answer | 25 | 7% | 25 | 7% | 50 | 7% |  |
|  | **Mean** |  | **Mean** |  | **Mean** |  |  |
| Age | 27.08 |  | 27.89 |  | 27.48 |  | 0.05 |
| Avg. years smartphone use | 6.5 |  | 6.6 |  | 6.5 |  | 0.80 |

Note: p-values from Chi-Square tests of independence of categorical variables, or from t-tests of equality for continuous variables.

**Table SM13.** Description of the treated and control sub-samples characteristics for the nudge based on disclosing the collectively produced set of prices for the (a) individuals that submitted prices at any point during the period of analysis of this nudge (pre-, during- and post-intervention) and (b) those that not.

|  | **Nudge 2: Disclosing collective data – (a) Active volunteers** | | | | | | |
| --- | --- | --- | --- | --- | --- | --- | --- |
|  | **Dashboard** | | **Control** | | **Full sample** | | **P-value** |
| **Variable** | **n** | **%** | **n** | **%** | **n** | **%** |  |
| Total | 124 | 100% | 120 | 100% | 244 | 100% |  |
| Gender |  |  |  |  |  |  | 0.29 |
| Female | 19 | 15% | 13 | 11% | 32 | 13% |  |
| Male | 105 | 85% | 107 | 89% | 212 | 87% |  |
| Education |  |  |  |  |  |  | 0.77 |
| Primary | 0 | 0% | 0 | 0% | 0 | 0% |  |
| Secondary | 17 | 14% | 18 | 15% | 35 | 14% |  |
| Tertiary | 107 | 86% | 102 | 85% | 209 | 86% |  |
| Food chain stage |  |  |  |  |  |  | 0.23 |
| Farmer | 55 | 44% | 69 | 58% | 124 | 51% |  |
| Final consumer | 39 | 31% | 34 | 28% | 73 | 30% |  |
| Retailer and wholesaler | 13 | 10% | 7 | 6% | 20 | 8% |  |
| Other | 17 | 14% | 10 | 8% | 27 | 11% |  |
| Preferred communication channel |  |  |  |  |  |  | 0.89 |
| App | 15 | 12% | 11 | 9% | 26 | 11% |  |
| Email | 19 | 15% | 19 | 16% | 38 | 16% |  |
| SMS | 75 | 60% | 74 | 62% | 149 | 61% |  |
| Web | 3 | 2% | 5 | 4% | 8 | 3% |  |
| Unknown | 12 | 10% | 11 | 9% | 23 | 9% |  |
| Nudge 1 |  |  |  |  |  |  | 0.36 |
| Treated | 56 | 45% | 52 | 43% | 108 | 44% |  |
| Control | 55 | 44% | 48 | 40% | 103 | 42% |  |
| Non participant | 13 | 10% | 20 | 17% | 33 | 14% |  |
| Personal motivation |  |  |  |  |  |  | 0.93 |
| Yes | 49 | 40% | 48 | 40% | 97 | 40% |  |
| No | 75 | 60% | 72 | 60% | 147 | 60% |  |
| Reward motivation |  |  |  |  |  |  | 0.12 |
| Yes | 49 | 40% | 59 | 49% | 108 | 44% |  |
| No | 75 | 60% | 61 | 51% | 136 | 56% |  |
| Data motivation |  |  |  |  |  |  | 0.23 |
| Yes | 101 | 81% | 102 | 85% | 203 | 83% |  |
| No | 11 | 9% | 7 | 6% | 18 | 7% |  |
| No answer | 12 | 10% | 11 | 9% | 23 | 9% |  |
| Market transactor |  |  |  |  |  |  | 0.12 |
| Yes | 6 | 5% | 2 | 2% | 8 | 3% |  |
| No | 110 | 89% | 103 | 86% | 213 | 87% |  |
|  | 8 | 6% | 15 | 13% | 23 | 9% |  |
|  | Mean |  | Mean |  | Mean |  |  |
| Age | 27.16 |  | 28.23 |  | 27.71 |  | 0.05* |
| Avg. years smartphone use | 6.19 |  | 6.29 |  | 6.24 |  | 0.72 |

Note: p-values from Chi-Square tests of independence between categorical variables, or from t-tests of equality for continuous variables.

|  | **Nudge 2: Disclosing collective data – (b) Non-active volunteers** | | | | | | |
| --- | --- | --- | --- | --- | --- | --- | --- |
|  | **Dashboard** | | **Control** | | **Full sample** | | **P-value** |
| **Variable** | **n** | **%** | **n** | **%** | **n** | **%** |  |
| Total | 248 | 100% | 245 | 100% | 493 | 100% |  |
| Gender |  |  |  |  |  |  | 0.67 |
| Female | 42 | 17% | 38 | 16% | 80 | 16% |  |
| Male | 206 | 83% | 207 | 84% | 413 | 84% |  |
| Education |  |  |  |  |  |  | 0.55 |
| Primary | 1 | 0% | 1 | 0% | 2 | 0% |  |
| Secondary | 32 | 13% | 34 | 14% | 66 | 13% |  |
| Tertiary | 215 | 87% | 210 | 86% | 425 | 86% |  |
| Food chain stage |  |  |  |  |  |  | 0.02** |
| Farmer | 96 | 39% | 98 | 38% | 194 | 39% |  |
| Final consumer | 99 | 40% | 85 | 33% | 184 | 37% |  |
| Retailer and wholesaler | 28 | 11% | 39 | 15% | 67 | 14% |  |
| Other | 25 | 10% | 39 | 15% | 48 | 10% |  |
| Preferred communication channel |  |  |  |  |  |  | 0.88 |
| App | 16 | 6% | 17 | 7% | 33 | 7% |  |
| Email | 48 | 19% | 51 | 21% | 99 | 20% |  |
| SMS | 161 | 65% | 157 | 64% | 318 | 65% |  |
| Web | 10 | 4% | 6 | 2% | 16 | 3% |  |
| Unknown | 13 | 5% | 14 | 6% | 27 | 5% |  |
| Nudge 1 |  |  |  |  |  |  | 0.35 |
| Treated | 44 | 18% | 39 | 16% | 83 | 17% |  |
| Control | 50 | 20% | 39 | 16% | 83 | 17% |  |
| Non participant | 154 | 62% | 173 | 69% | 327 | 66% |  |
| Personal motivation |  |  |  |  |  |  | 0.46 |
| Yes | 92 | 37% | 83 | 34% | 175 | 35% |  |
| No | 156 | 63% | 162 | 66% | 318 | 65% |  |
| Reward motivation |  |  |  |  |  |  | 0.63 |
| Yes | 90 | 36% | 94 | 38% | 184 | 37% |  |
| No | 158 | 64% | 151 | 62% | 309 | 63% |  |
| Data motivation |  |  |  |  |  |  | 0.58 |
| Yes | 208 | 84% | 211 | 86% | 419 | 85% |  |
| No | 27 | 11% | 20 | 8% | 47 | 10% |  |
| No answer | 13 | 5% | 14 | 6% | 27 | 5% |  |
| Market transactor |  |  |  |  |  |  | 0.38 |
| Yes | 1 | 0% | 0 | 0% | 1 | 0% |  |
| No | 93 | 38% | 72 | 29% | 165 | 33% |  |
|  | 154 | 62% | 173 | 71% | 327 | 66% |  |
|  | Mean |  | Mean |  | Mean |  |  |
| Age | 27.04 |  | 27.7 |  |  |  | 0.2 |
| Avg. years smartphone use | 6.7 |  | 6.71 |  |  |  | 0.94 |

Note: p-values from Chi-Square tests of independence between categorical variables, or from t-tests of equality for continuous variables.

**Table SM14.** The effects of price disclosure on the **number of weekly price submissions** by a) active and b) non-active volunteers, comparing pre-intervention (Weeks 21-27) with intervention period (Weeks 28-34).

|  | 1. Active | | | 1. Non-active | | |
| --- | --- | --- | --- | --- | --- | --- |
| VARIABLES | Model 1 | Model 2 | Model 3 | Model 1 | Model 2 | Model 3 |
|  |  |  |  |  |  |  |
| Time#Treated (DID) | -5.08 | -4.13 | -1.42 | -4.30 | -4.20 | -4.05 |
|  | (3.92) | (3.88) | (3.98) | (8.49) | (8.56) | (8.60) |
| Time | 8.28*** | 6.51** | 4.56* | 7.48** | 5.97* | 19.3 |
|  | (3.03) | (2.82) | (2.71) | (3.46) | (3.22) | (14.3) |
| Treated | -0.82 | -0.21 | 0.34 | 5.25 | 5.25 | 4.83 |
|  | (4.32) | (4.07) | (3.89) | (5.50) | (5.56) | (4.61) |
| Reward (previous week) |  | 16.5*** | 16.6*** |  | 5.68* | 0.83 |
|  |  | (2.70) | (2.72) |  | (2.90) | (4.27) |
| Nudge1 |  |  | 10.8** |  |  | 1.54 |
|  |  |  | (5.33) |  |  | (12.3) |
| Gender (=1, male) |  |  | 3.43 |  |  | 20.1 |
|  |  |  | (7.42) |  |  | (13.5) |
| Age (=1, <30) |  |  | 4.80 |  |  | 10.1 |
|  |  |  | (5.16) |  |  | (14.1) |
| Education (=1, tertiary education) |  |  | 5.58 |  |  | - |
|  |  |  | (6.31) |  |  |  |
| Preferred communication method (=1, SMS) |  |  | 16.3*** |  |  | 7.05 |
|  |  |  | (4.38) |  |  | (8.32) |
| Farmer |  |  | 11.3* |  |  | -0.13 |
|  |  |  | (6.77) |  |  | (3.79) |
| Consumer |  |  | 12.4* |  |  | -5.14 |
|  |  |  | (6.80) |  |  | (4.74) |
| Trader |  |  | 22.3 |  |  | 16.3** |
|  |  |  | (17.3) |  |  | (6.48) |
| Motivation to participate personal (=1, yes) |  |  | 0.72 |  |  | -23.4*** |
|  |  |  | (5.77) |  |  | (4.59) |
| Motivation to participate reward (=1, yes) |  |  | -4.07 |  |  | -1.88 |
|  |  |  | (5.97) |  |  | (12.8) |
| Motivation to participate data (=1, yes) |  |  | 9.50* |  |  | - |
|  |  |  | (5.53) |  |  |  |
| Constant | 29.7*** | 22.4*** | -24.7 | 10.2** | 10.2** | -24.0* |
|  | (2.95) | (2.60) | (18.3) | (4.35) | (4.39) | (13.2) |
|  |  |  |  |  |  |  |
| Observations | 1,475 | 1,475 | 1,301 | 52 | 52 | 35 |
| R-squared | 0.011 | 0.073 | 0.178 | 0.015 | 0.034 | 0.654 |

OLS regressions, robust clustered errors at the volunteer ID level in parentheses

*** p<0.01, ** p<0.05, * p<0.1

**Table SM15.** The effects of price disclosure on the **number of weekly price submissions** by volunteers, comparing pre-intervention (Weeks 21-27) with intervention period (Weeks 28-34), controlling for whether the nudge goes to the a) market actors or b) market observers.

|  | 1. Market actor | | | 1. Market observer | | |
| --- | --- | --- | --- | --- | --- | --- |
| VARIABLES | Model 1 | Model 2 | Model 3 | Model 1 | Model 2 | Model 3 |
|  |  |  |  |  |  |  |
| Time#Treated (DID) | -5.12 | -4.46 | 1.02 | -4.28 | -3.35 | -0.78 |
|  | (5.80) | (5.70) | (7.15) | (4.09) | (4.06) | (3.93) |
| Time | 7.65 | 6.01 | 2.98 | 6.02* | 4.77 | 2.64 |
|  | (4.68) | (4.32) | (4.72) | (3.17) | (2.99) | (2.51) |
| Treated | 6.22 | 6.12 | 8.86 | -7.26 | -6.22 | -3.72 |
|  | (7.09) | (6.68) | (6.32) | (4.79) | (4.49) | (4.94) |
| Reward (previous week) |  | 18.4*** | 16.5*** |  | 14.3*** | 14.1*** |
|  |  | (4.49) | (3.68) |  | (2.43) | (2.36) |
| Nudge1 |  |  | 7.56 |  |  | 13.2** |
|  |  |  | (7.42) |  |  | (5.25) |
| Gender (=1, male) |  |  | -6.00 |  |  | 0.72 |
|  |  |  | (9.51) |  |  | (5.56) |
| Age (=1, <30) |  |  | 3.00 |  |  | 1.36 |
|  |  |  | (8.06) |  |  | (5.36) |
| Education (=1, tertiary education) |  |  | -0.57 |  |  | 5.14 |
|  |  |  | (9.16) |  |  | (6.59) |
| Preferred communication method (=1, SMS) |  |  | 18.2** |  |  | 10.5** |
|  |  |  | (8.05) |  |  | (4.51) |
| Farmer |  |  | 24.4** |  |  | 4.81 |
|  |  |  | (11.0) |  |  | (5.43) |
| Consumer |  |  | 27.8*** |  |  | 2.42 |
|  |  |  | (10.0) |  |  | (7.42) |
| Trader |  |  | 58.3* |  |  | 2.35 |
|  |  |  | (31.7) |  |  | (6.80) |
| Motivation to participate personal (=1, yes) |  |  | 12.9 |  |  | -5.86 |
|  |  |  | (9.95) |  |  | (5.64) |
| Motivation to participate reward (=1, yes) |  |  | -9.83 |  |  | -3.10 |
|  |  |  | (8.95) |  |  | (6.54) |
| Motivation to participate data (=1, yes) |  |  | 25.6* |  |  | 3.98 |
|  |  |  | (14.5) |  |  | (6.79) |
| Constant | 27.3*** | 19.4*** | -43.5 | 31.5*** | 25.2*** | 0.59 |
|  | (4.58) | (3.56) | (29.5) | (3.70) | (3.53) | (10.6) |
|  |  |  |  |  |  |  |
| Observations | 761 | 761 | 669 | 766 | 766 | 667 |
| R-squared | 0.008 | 0.067 | 0.234 | 0.039 | 0.111 | 0.242 |

OLS regressions, robust clustered errors at the volunteer ID level in parentheses

*** p<0.01, ** p<0.05, * p<0.1

**Table SM16.** The effects of price disclosure on the **percentage of valid weekly** price submissions by volunteers, comparing pre-intervention (Weeks 21-27) with (a) intervention period (Weeks 28-34) and (b) post-treatment period (Weeks 35-37).

|  | 1. Pre-intervention vs intervention | | | 1. Pre-intervention vs post-intervention | | |
| --- | --- | --- | --- | --- | --- | --- |
| VARIABLES | Model 1 | Model 2 | Model 3 | Model 1 | Model 2 | Model 3 |
|  |  |  |  |  |  |  |
| Time#treated (DID) | 0.6 | 0.6 | 0.6 | -1.5 | -1.6 | -1.4 |
|  | (1.4) | (1.4) | (1.5) | (1.6) | (1.6) | (1.8) |
| Time | -1.0 | -0.9 | -1.0 | 2.7** | 2.7** | 2.2* |
|  | (1.0) | (1.0) | (1.1) | (1.1) | (1.1) | (1.3) |
| Treated | -0.1 | -0.2 | 0.2 | -0.1 | -0.1 | -0.004 |
|  | (1.4) | (1.4) | (1.4) | (1.4) | (1.4) | (1.4) |
| Reward (previous week) |  | -0.7 | -0.7 |  | 0.5 | 0.7 |
|  |  | (0.8) | (0.9) |  | (0.9) | (1.0) |
| Nudge1 |  |  | -1.7 |  |  | -1.2 |
|  |  |  | (1.4) |  |  | (1.3) |
| Gender (=1, male) |  |  | 0.8 |  |  | -1.5 |
|  |  |  | (2.2) |  |  | (1.9) |
| Age (=1, <30) |  |  | -3.8** |  |  | -3.5** |
|  |  |  | (1.5) |  |  | (1.4) |
| Education (=1, tertiary education) |  |  | -1.5 |  |  | -2.2 |
|  |  |  | (2.1) |  |  | (2.1) |
| Preferred communication method (=1, SMS) |  |  | -2.8* |  |  | -3.1** |
|  |  |  | (1.5) |  |  | (1.4) |
| Farmer |  |  | 0.04 |  |  | 0.05 |
|  |  |  | (2.6) |  |  | (2.0) |
| Consumer |  |  | 2.4 |  |  | 1.8 |
|  |  |  | (2.8) |  |  | (2.3) |
| Trader |  |  | -0.4 |  |  | 0.6 |
|  |  |  | (2.8) |  |  | (2.2) |
| Motivation to participate personal (=1, yes) |  |  | 0.2 |  |  | -1.3 |
|  |  |  | (1.4) |  |  | (1.4) |
| Motivation to participate reward (=1, yes) |  |  | 2.7* |  |  | 1.7 |
|  |  |  | (1.4) |  |  | (1.4) |
| Motivation to participate data (=1, yes) |  |  | -5.1*** |  |  | -3.3** |
|  |  |  | (1.6) |  |  | (1.5) |
| Constant | 91*** | 91*** | 100*** | 91*** | 91*** | 101*** |
|  | (1.0) | (1.1) | (4.4) | (1.0) | (1.2) | (4.2) |
|  |  |  |  |  |  |  |
| Observations | 1,477 | 1,477 | 1,291 | 1,090 | 1,090 | 946 |
| R-squared | 0.001 | 0.001 | 0.062 | 0.006 | 0.006 | 0.060 |

OLS regressions, robust clustered standard errors at the volunteer ID level in parentheses.

*** p<0.01, ** p<0.05, * p<0.1

**Table SM17.** Average percentage of **valid price submissions** by volunteers in a given week before, during and after the intervention based on the communication of crowdsourced prices (nudge 2) – standard errors in parentheses.

|  | Pre-intervention | Intervention | Post-intervention |
| --- | --- | --- | --- |
| All | 90.82  (0.47) | 90.10  (0.52) | 91.92  (1.05) |
| Treated | 90.75  (0.66) | 90.33  (0.76) | 93.59  (0.80) |
| Control | 90.88  (0.68) | 89.89  (0.71) | 93.59  (0.79) |


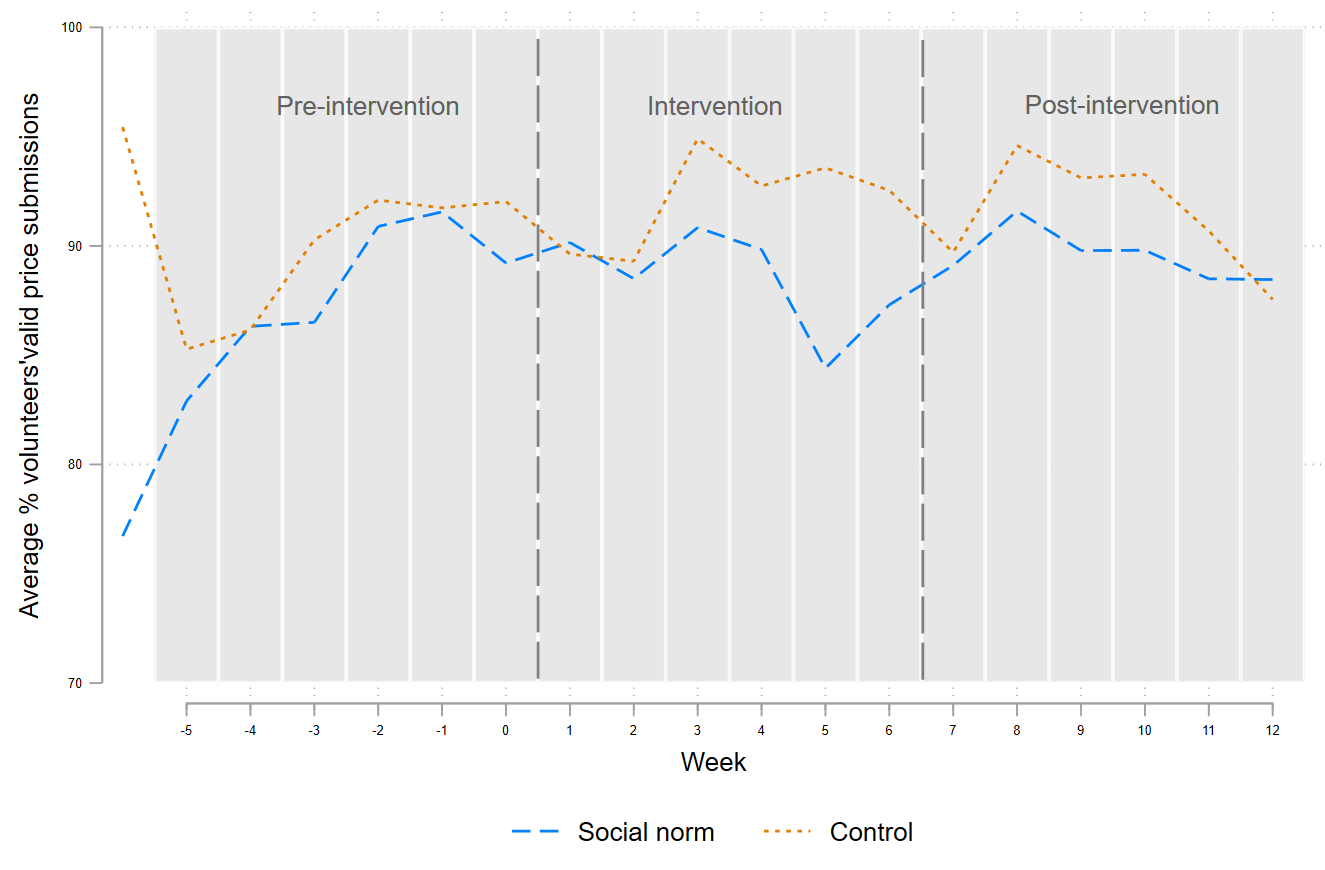
**Figure SM2.** Average percentage of valid volunteers’ weekly submissions within the nudge (nudge 1) and control groups.


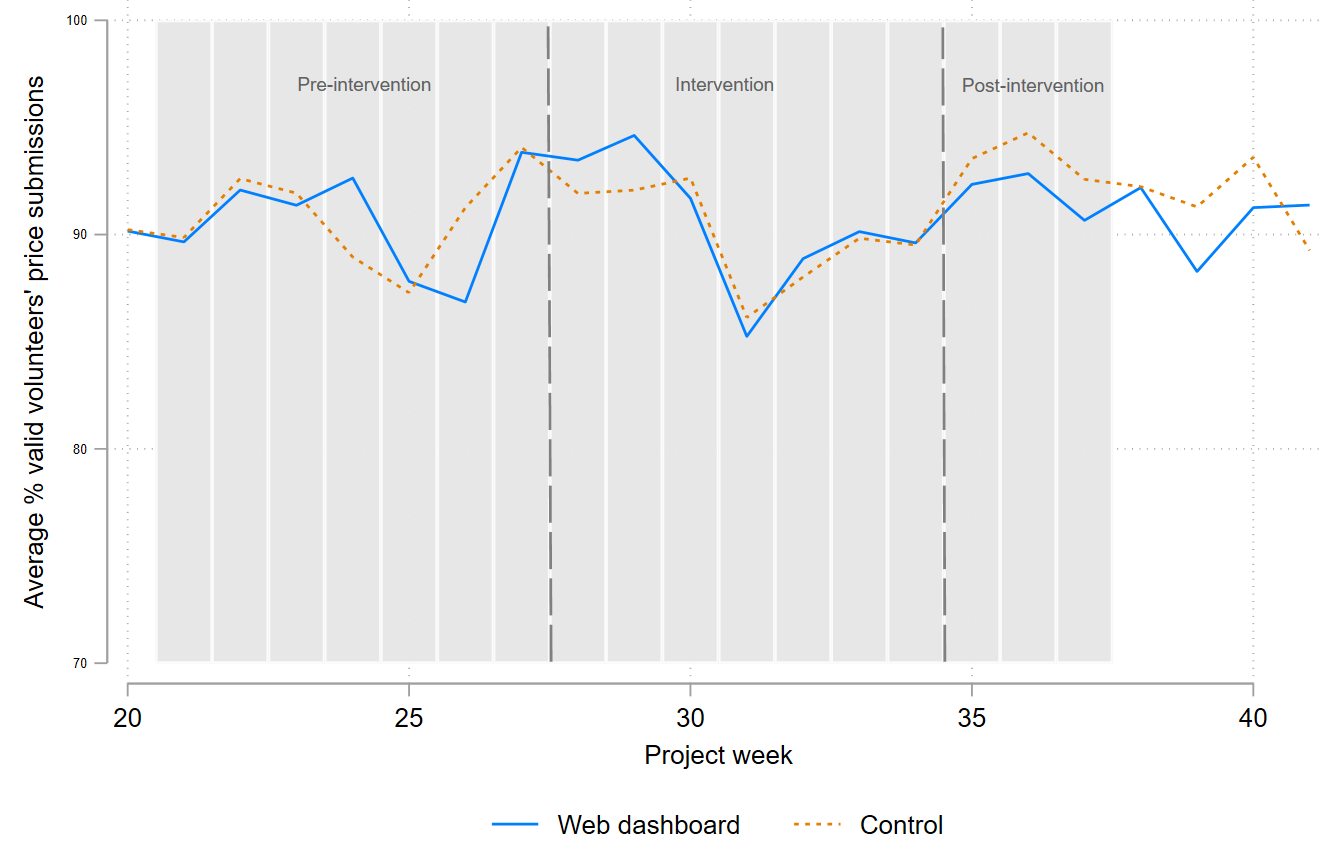
**Figure SM3.** Average percentage of valid volunteers’ weekly submissions within the nudge (nudge 2) and control groups.
